# Protein retention in the endoplasmic reticulum rescues Aβ toxicity in *Drosophila*^☆^

**DOI:** 10.1016/j.neurobiolaging.2023.09.008

**Published:** 2023-12

**Authors:** James H. Catterson, Lucy Minkley, Salomé Aspe, Sebastian Judd-Mole, Sofia Moura, Miranda C. Dyson, Arjunan Rajasingam, Nathaniel S. Woodling, Magda L. Atilano, Mumtaz Ahmad, Claire S. Durrant, Tara L. Spires-Jones, Linda Partridge

**Affiliations:** a Institute of Healthy Ageing, Genetics, Evolution and Environment, University College London, Darwin Building, Gower Street, London WC1E 6BT, UK; b Centre for Discovery Brain Sciences, UK Dementia Research Institute, The University of Edinburgh, 1 George Square, Edinburgh EH8 9JZ, Scotland, UK; c Max Planck Institute for Biology of Ageing, Joseph-Stelzmann-Strasse 9b, 50931 Cologne, Germany

**Keywords:** Alzheimer’s disease, Laminin, *Drosophila melanogaster*, Aβ toxicity, Endoplasmic reticulum, ER retention

## Abstract

Amyloid β (Aβ) accumulation is a hallmark of Alzheimer’s disease. In adult *Drosophila* brains, human Aβ overexpression harms climbing and lifespan. It’s uncertain whether Aβ is intrinsically toxic or activates downstream neurodegeneration pathways. Our study uncovers a novel protective role against Aβ toxicity: intra-endoplasmic reticulum (ER) protein accumulation with a focus on laminin and collagen subunits. Despite high Aβ, laminin B1 (LanB1) overexpression robustly counters toxicity, suggesting a potential Aβ resistance mechanism. Other laminin subunits and collagen IV also alleviate Aβ toxicity; combining them with LanB1 augments the effect. Imaging reveals ER retention of LanB1 without altering Aβ secretion. LanB1’s rescue function operates independently of the IRE1α/XBP1 ER stress response. ER-targeted GFP overexpression also mitigates Aβ toxicity, highlighting broader ER protein retention advantages. Proof-of-principle tests in murine hippocampal slices using mouse Lamb1 demonstrate ER retention in transduced cells, indicating a conserved mechanism. Though ER protein retention generally harms, it could paradoxically counter neuronal Aβ toxicity, offering a new therapeutic avenue for Alzheimer’s disease.

## Graphical abstract

**Figure:**
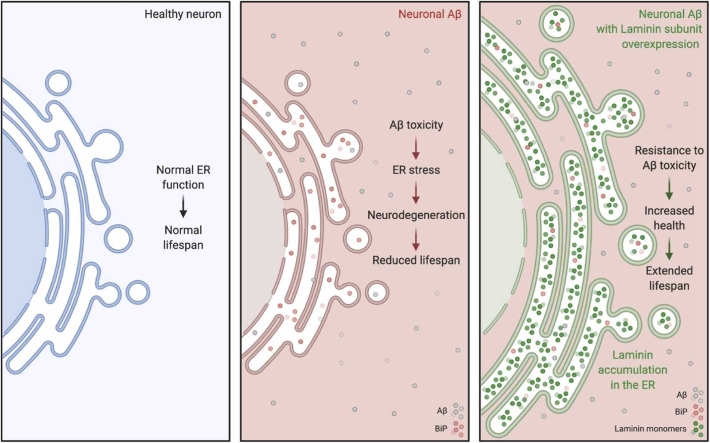

## 1 Introduction

Despite decades of intensive research and a large number of clinical trials, Alzheimer’s disease (AD) has no cure. Most cases are sporadic, with age being the biggest risk factor. The neuropathological hallmarks include the accumulation of Amyloid β (Aβ) plaques and neurofibrillary tau tangles (Huang and Mucke, 2012). AD is thought to be triggered by the accumulation of Aβ peptides, derived from the misprocessing of amyloid precursor protein (APP), resulting in increased cellular stress, accumulation of toxic hyperphosphorylated tau, and eventual neuronal cell death (Hardy and Higgins, 1992). Characteristic features of AD include aggregates of unfolded proteins, increased reactive oxygen species, and metabolic dysregulation in the affected neurons (Gerakis and Hetz, 2017, Hetz and Mollereau, 2014). The endoplasmic reticulum (ER) recognizes these alterations in neuronal homeostasis, and consequently, AD brains display many signs of ER stress (Hamos et al., 1991, Hoozemans et al., 2009), which appears early in AD progression (Cornejo and Hetz, 2013, Unterberger et al., 2006). ER stress generally occurs upon the accumulation of unfolded or misfolded proteins in the ER lumen. The ER chaperone BiP (Grp78) dissociates from membrane sensors (inositol-requiring enzyme 1α [IRE1α], protein kinase-like endoplasmic reticulum kinase, and activating transcription factor 6) and binds to these misfolded proteins (Bertolotti et al., 2000). With the removal of BiP, IRE1α oligomerizes then catalyzes the “unconventional” splicing of X-box binding protein 1 (Xbp1) mRNA, generating XBP1s, a transcription factor that regulates genes involved in the ER stress response, including BiP (Uddin et al., 2020).

Aβ pathology is largely extracellular, but there is also a lively debate (Bowman Rogers, 2015, Strobel, 2011) in the AD field about intracellular Aβ, which may play a major role in neurodegeneration (Ji et al., 2016, Takahashi et al., 2017). Although the existence of intracellular Aβ has become less controversial, its role in the induction of the ER stress response is unresolved, and the mechanisms by which protein misfolding contributes to AD pathogenesis are still unclear (Scheper and Hoozemans, 2015).

The detailed molecular mechanisms underlying the etiology of AD remain to be confirmed, and many *in vitro* and *in vivo* models have been developed to study them (Fernandez-Funez et al., 2015, Gama Sosa et al., 2011). *Drosophila melanogaster* has been widely used as a model system to study neurodegenerative disorders, including AD (Fernandez-Funez et al., 2015). In the adult fly brain, neuronally expressed Aβ is predominantly found in the cell body of neurons but can also be observed in the neuropil when glial function is affected (Ray et al., 2017), indicating that glia, which accounts for ∼10% of the cells (Kremer et al., 2017) yet cover a large area of the adult fly brain (Awasaki et al., 2008), play an important role clearing Aβ.

In flies, the ER stress response is activated in response to neuronal Aβ, with increased Xbp1 splicing and BiP levels (Casas-Tinto et al., 2011, Marcora et al., 2017, Niccoli et al., 2016). Indeed, Xbp1 acts to buffer the toxic effects of Aβ since overexpression of spliced Xbp1 reduces Aβ levels and partially rescues toxicity, while Xbp1 knockdown increases Aβ levels and exacerbates toxicity (Casas-Tinto et al., 2011, Marcora et al., 2017).

The ER is involved in the secretion of many proteins, including those targeted to the extracellular milieu, such as collagens and laminins (Rios-Barrera et al., 2017). The assembly of laminins and collagens from their individual subunits occurs in the ER before secretion into the trans-Golgi network and then toward their final destination outside the cell, where they form the extracellular matrix (ECM) (Liu et al., 2017). Laminins are obligate heterotrimers consisting of large α, β, and γ subunits that combine via the triple-helical coiled-coil domain in the center of each chain to form cruciform-shaped structures. The expression of the β1 subunit of the ECM protein Laminin (LamB1) appears restricted to regions of the brain that are susceptible to neurodegeneration, especially the hippocampal tri-synaptic circuit (Indyk et al., 2003, Sharif et al., 2004). Indeed, modulating LamB1 expression has been associated with changes in memory formation (Yang et al., 2011), while Aβ-induced memory deficits are rescued by LamB1 modulation (Hsu et al., 2013). Under normal conditions, laminin interacts with APP (Kibbey et al., 1993). Additionally, antilaminin immunoreactivity levels in human cerebrospinal fluid have been shown to correlate with the pathogenesis of AD and vascular dementia (Matsuda et al., 2002). *In vitro*, the laminin heterotrimer can inhibit Aβ fibrillation and depolymerize Aβ, although without reduction of toxicity (Bronfman et al., 1996, Monji et al., 1999, Monji et al., 1998). Notably, Aβ can induce memory deficits that can be rescued by ECM manipulation (Cheng et al., 2009, Hsu et al., 2013, Lane-Donovan et al., 2015, Végh et al., 2014). The complexity of neuron-matrix interactions makes it difficult to recapitulate ECM organization and function in cell culture. It is, therefore, important to design experiments to evaluate these interactions *in vivo*.

Laminin in the brain is typically found extracellularly at basement membranes in the vasculature and at the blood-brain barrier (Xu et al., 2018). However, synaptic laminin has also been characterized at the neuromuscular junction (Patton, 2000; Tsai et al., 2012). In the hippocampus, synaptic α5-laminin was found to be necessary and sufficient to stabilize dendritic spine dynamics (Omar et al., 2017). Surprisingly, intraneuronal laminin expression has also been identified in the hippocampus and may have functions other than extracellular structural integrity (Chen et al., 2003, Yang et al., 2011). Indeed, laminin has been observed in hippocampal neuronal perikarya and other susceptible regions of the brain important in the development of AD (Brandan and Inestrosa, 1993, Yamamoto et al., 1988). Laminin expression was also observed to be increased in the AD prefrontal cortex compared to nondisease controls (Palu and Liesi, 2002). Similarly, the collagen VI α1 chain was upregulated in dentate gyrus soma in mice expressing hAPP and could rescue Aβ toxicity *in vitro* by sequestering Aβ into large aggregates in the extracellular milieu (Cheng et al., 2009).

**Fig. 1:**
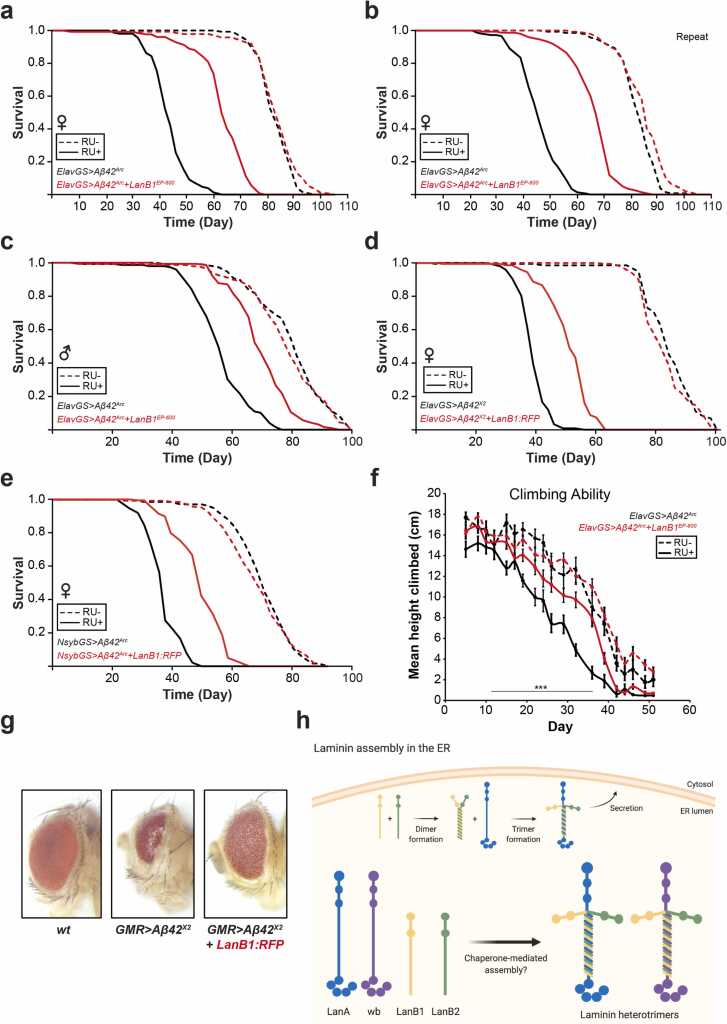
**Co-expression of LanB1 rescued the toxic effect of Aβ expression**. (A) Survival curves of female flies expressing Aβ^Arc^ in adult neurons. Induction of Aβ^Arc^ with Elav-GeneSwitch significantly (*p* = 1.31 × 10^−70^; log-rank test) shortened lifespan compared to uninduced controls. LanB1 and Aβ^Arc^ co-expression resulted in a significant rescue (*p* = 1.38 × 10^−50^; log-rank test) of the short-lived phenotype. (B) Repeat of experiment in (A). LanB1 significantly rescued Aβ^Arc^ toxicity (*p* = 8.31 × 10^−56^; log-rank test). (C) LanB1 significantly rescued Aβ^Arc^ toxicity in male flies (*p* = 8.31 × 10^−56^; log-rank test). (D) Induction of Aβ^X2^ significantly (*p* = 5.99 × 10^−69^; log-rank test) shortened lifespan compared to uninduced controls. LanB1 and Aβ^X2^ co-expression resulted in a significant rescue (*p* = 8.04 × 10^−40^; log-rank test). (E) Induction of Aβ^Arc^ with NsybGS significantly (*p* = 3.38 × 10^−67^; log-rank test) shortened lifespan compared to uninduced controls. LanB1 significantly rescued Aβ^Arc^ toxicity (*p* = 6.03 × 10^−40^; log-rank test). Dashed lines represent uninduced “RU−” controls, and solid lines represent induced “RU+” conditions. For all lifespan experiments, n = 150 flies per condition. (F) Climbing ability was measured until day 51. Climbing ability of Aβ^Arc^ flies was lower than that of all other groups (*p* < 0.0001, 2-way analysis of variances with Tukey’s post-hoc test). LanB1 significantly rescued the Aβ^Arc^-induced decline in climbing ability, though not to the same level as uninduced controls (*p* < 0.0001, 2-way analysis of variances with Tukey’s post-hoc test). Data are shown as mean ± SEM (n = 37–70 flies measured per time point). (G) LanB1 suppressed Aβ toxicity in the eyes of flies raised at 29 °C. Control flies displayed a highly ordered ommatidia lattice (left). Expression of Aβ^X2^ using the eye-specific GMR-GAL4 driver resulted in small, glassy eyes that accumulated necrotic spots (middle). Co-expression of Aβ^X2^ with LanB1 rescued most of the size and organization defects (right). (H) *Drosophila* laminins and their assembly in the ER. Created using BioRender. Abbreviations: Aβ, amyloid β; ER, endoplasmic reticulum; ElavGS, Elav-GeneSwitch; LanB1, Laminin B1.

Vertebrates have multiple genes for all 3 laminin subunits; 5 α-chains (α1–α5), 4 β-chains (β1–β4), and 3 γ-chains (γ1–γ3) are known, and they combine to form at least 16 different laminin heterotrimers (Domogatskaya et al., 2012), not including novel spliced forms (Hamill et al., 2009). In *Drosophila*, there are 2 α (LanA and wb), 1 β (LanB1), and 1 γ (LanB2) subunits, resulting in only 2 laminin heterotrimers (Urbano et al., 2009) (Fig. 1H). Laminin trimerization occurs in the ER where the β and γ subunits assemble and require α subunit incorporation to form a functional laminin heterotrimer before secretion (Kumagai et al., 1997, Morita et al., 1985, Yurchenco et al., 1997). If either the β or γ subunit is missing, the other accumulates intracellularly, and the α subunit can be secreted as a monomer. Knockdown of α laminin also results in β and γ subunit retention in the ER (De Arcangelis et al., 1996). Similarly, overexpression of laminin monomers in glial cells leads to ER expansion and triggers ER stress, impairing correct development and locomotion in *Drosophila* larvae (Petley-Ragan et al., 2016). Collagen IV is another obligate heterotrimer that can accumulate intracellularly when subunit stoichiometry is altered (Boot-Handford and Briggs, 2009).

If ER stress is part of the AD pathogenic cascade, and it is also activated in response to modulation of laminin/collagen subunit stoichiometry, then experimentally increasing ectopic ECM protein subunit expression in adult neurons might be expected to overwhelm the ER and exacerbate AD pathogenesis. On the contrary, we found that neuronal overexpression of *Drosophila* laminin and collagen subunits led to pronounced intra-ER accumulation of these subunits, yet could robustly ameliorate the toxic effects of Aβ, such as reduced lifespan and climbing ability, without reducing Aβ levels. We also found that overexpression of mouse Lamb1 in murine organotypic hippocampal slice cultures resulted in ER retention of these monomers, indicating a conserved mechanism. Interestingly, overexpression of ER-targeted GFP also rescued Aβ toxicity, indicating a potentially broader benefit of ER protein retention. Protein accumulation in the ER is typically seen as detrimental to cellular health, but in the context of neuronal Aβ toxicity, it may prove to be beneficial.

## 2 Methods

### 2.1 *Drosophila* stocks and fly husbandry

The wild-type stock Dahomey was collected in 1970 in Dahomey (now Benin) and has since been maintained in large population cages with overlapping generations on a 12L:12D cycle at 25 °C. The white Dahomey (w^Dah^) stock was derived by the incorporation of the w^1118^ deletion into the outbred Dahomey background by successive backcrossing. The UAS-(G_4_C_2_)_36_ line was generated in our lab (Mizielinska et al., 2014).

The following stocks were obtained from the Bloomington *Drosophila* Stock Center: UAS-LanB1^EP-600^ (#43428), UAS-LanA^EY02207^ (#20141), UAS-cdc14^EY10303^ (#16450), UAS-Col13A1^EY09983^ (#17628), Glass Multimer Reporter-GAL4 (GMR-GAL4) (#9146), UAS-polyQ(Q108) (#68393), UAS-GFP (#1521), UAS-H3:GFP (#68241), UAS-mCD8:GFP (#5137), UAS-mCD8:RFP (#27392), and UAS-KDEL:GFP (#9898). The following stocks were obtained from the Vienna *Drosophila* Resource Center: UAS-Mys^VDRC^ (β1-integrin) (#29619), UAS-Mew^VDRC^ (αPS1-integrin) (#44890), UAS-wb^VDRC^ (wb^RNAi 1^) (#3141), and UAS-Xbp1^VDRC^ (#109312). The following stocks were obtained from the Transgenic RNAi Project at Harvard Medical School: UAS-mCherry^TRiP^ (#35785), UAS-LanA^TRiP^ (#28071), UAS-LanB2^TRiP^ (LanB2^RNAi 1^) (#55388), UAS-LanB2^TRiP^ (LanB2^RNAi 2^) (#62002), and UAS-wb^TRiP^ (wb^RNAi 2^) (#29559). UAS-Aβ_42_^Arctic^ was a gift from Dr Damian Crowther (University of Cambridge, UK) (Crowther et al., 2005). UAS-Aβ_42_^X2^ (tandem wild-type Aβ) was a gift from Dr Pedro Fernandez-Funez (University of Minnesota, USA) (Casas-Tinto et al., 2011). UAS-Mys (β1-integrin) was a gift from Dr Rongwen Xi (NIBS, China) (Lin et al., 2013). UAS-LanB1:RFP, UAS-Cg25C:RFP, UAS-LanB2:GFP, and UAS-secr:GFP (SP^Wg^.GFP) lines were a gift from Dr José Carlos Pastor-Pareja (Tsinghua University, China) (Ke et al., 2018; Liu et al., 2017). Elav-GeneSwitch (ElavGS) driver line was a gift from Dr Hervé Tricoire (CNRS, France) (Latouche et al., 2007). NsybGS driver line was a gift from Dr Amita Sehgal (UPenn, USA) (Bai et al., 2018). GliaGS driver line (GSG3285-1) was a gift from Dr Minoru Saitoe (TMiMS, Japan) originally generated by Dr Haig Keshishian (Yale, USA) (Nicholson et al., 2008). Dilp2-GS driver line (stock #3 on the second chromosome) was a gift from Dr Heinrich Jasper (Buck Institute, USA) (Karpac et al., 2009).

Mutants and transgenic lines were backcrossed into the w^Dah^ *Wolbachia*-positive strain for at least 6 generations. Fly stocks were maintained and all experiments were conducted at 25 °C on a 12 h:12 h light/dark circadian cycle at constant 65% humidity using standard sugar/yeast/agar medium (containing 10% [w/v] brewer’s yeast, 5% [w/v] sucrose, and 1.5% [w/v] agar) (Bass et al., 2007). For all experiments involving Mifepristone, the steroid drug inducer “RU” (RU486—Sigma, Poole, Dorset, UK), the compound was dissolved in EtOH to make 100 mM stock solution and added to molten fly food for a final concentration of 200 μM. RU food was then dispensed into plastic vials, allowed to cool, then sealed and stored in a 4 °C cold room. RU food was brought to room temperature before use with flies.

### 2.2 Lifespan assay

Flies were reared at a standard density before being used for lifespan experiments, as previously described (Bass et al., 2007; Clancy and Kennington, 2001). All experiments were performed with flies (females and males) that were allowed 48 hours to mate after emerging as adults. Flies were subsequently lightly anesthetized with CO_2_, sorted into single sexes and counted at 15 per vial into 10 vials for a total of 150 flies per condition. In all cases, flies were transferred to fresh food at least 3 times a week, at which point deaths/censors were scored. For some survival assays, vials were kept in DrosoFlippers (drosoflipper.com) for ease of regular transfer to fresh vials. Microsoft Excel (lifespan template available at piperlab.org/resources/) was used to calculate survival proportions. Log-rank tests of survivorship curves were performed in Excel (Microsoft), and Cox proportional hazards analysis for multiple comparisons was performed in R Studio (R Core Team).

### 2.3 Climbing assay

For each condition analyzed in climbing assays, 5 vials of control food and 5 vials of food containing RU, each containing 15 flies, were housed side-by-side in a single DrosoFlipper. Flies were maintained as in lifespan studies and then were filmed for climbing assays once to twice per week. For climbing assays, the flies were kept in DrosoFlippers and transferred to empty vials on each side of the flipper, creating a standard vertical column 20 cm in height for each set of flies. Flies were tapped to the bottom of the vials and allowed to climb upward for 15 seconds before a still camera image was captured. The heights of individual flies were then assessed by manual multipoint selection in Fiji software (Schindelin et al., 2012), with each height in pixels calibrated to a height in centimeters from a ruler placed next to the vials during filming.

### 2.4 *Drosophila* rough eye phenotype analysis

Virgin females expressing Aβ^X2^ under the control of the GMR-Gal4 driver were crossed to males of the relevant genotype. Before imaging, flies were briefly snap frozen in liquid nitrogen to aid in correct positioning and avoid any movement artifacts. Eye images of 7-day-old flies were taken using a Leica M165 FC stereomicroscope equipped with a motorized stage and a multifocus tool (Leica application suite software). For eye size analysis, wide-field images at the same magnification were taken of flies for each genotype and eye size was measured using Fiji (Schindelin et al., 2012).

### 2.5 Capillary feeder (CAFE) assay

A 7 mL bijou vial filled with 1 mL of (1%) agar, to ensure humid conditions, was sealed with Parafilm (Alpha Laboratories Ltd, Hampshire, UK). Four holes in the Parafilm that were equally spaced apart were made using a 26-gauge needle to ensure adequate air circulation. Through the Parafilm was inserted a truncated 200 μL pipette tip that held a graduated 5 μL disposable glass capillary tube (Camag, Muttenz, Switzerland) containing liquid food (2% [w/v] yeast and 5% [w/v] sugar) supplemented with blue food dye (Langdale, Market Harborough, UK) to aid measurement of feeding. For all experiments, a mineral oil overlay (0.1 μL) was used to minimize evaporation. Food ingestion was measured every 24 hours. Each experiment included an identical, CAFE chamber without flies to determine evaporative losses (typically 10% of ingested volumes), which were subtracted from experimental readings (Ja et al., 2007).

### 2.6 Brain dissection, immunohistochemistry, and imaging of the fly brain

We followed the dissection and staining protocol for Aβ detection in the adult *Drosophila* brain from Ray et al. (2017). Using forceps, fly heads were removed from the bodies in cold phosphate buffered saline (PBS) containing 4% paraformaldehyde (Pierce, Thermo Fisher) + 0.01% Triton X-100 (Sigma). The proboscis was then removed from the fly head to allow fixative and detergent to fix and permeabilize the fly brain. The fly heads were transferred to a 0.5 mL microcentrifuge tube, fixed in 400 μL 4% paraformaldehyde + 0.01% Triton X-100 and rocked for 16 minutes at room temperature. The heads were washed with PBST-1 (PBS containing 0.01% Triton X-100) for 3 × 2 minutes at room temperature. The brains were dissected from the fly heads in ice-cold PBST-1 in a dissection dish under a light stereomicroscope. The brains were transferred to a new 0.5 mL microcentrifuge tube, fixed in 400 μL 4% paraformaldehyde + 0.1% Triton X-100, and rocked for 20 minutes at room temperature. The brains were then washed in PBST-2 (PBS containing 0.1% Triton X-100) for 3 × 2 minutes and blocked with SeaBlock blocking buffer (Thermo Fisher) for 15 minutes at room temperature. SeaBlock blocking buffer was removed and the brains were incubated in primary antibody in PBST-2 overnight, washed in PBST-2 for 3 × 20 minutes and incubated in secondary antibody in PBST-2 for 1 hour in the dark. The brains were washed in PBST-2 for 3 × 20 minutes at room temperature in the dark and mounted on a microscope slide using Vectashield mounting media containing DAPI (Vector Labs). The following antibodies were used. Primary antibodies: mouse anti-Aβ 6E10 (1:500; BioLegend #803001). Secondary antibodies: Alexa Fluor 488 donkey anti-mouse (1:400; Thermo Fisher #A21202), Alexa Fluor 594 goat anti-mouse (1:400; Thermo Fisher #A11005). Images were captured with a Zeiss LSM 700 confocal laser scanning microscope (Zeiss, Germany) or with a Leica TCS8 confocal microscope (Leica, Wetzlar, Germany) with a 20× or 63× oil immersion objective. Images were taken as stacks and are shown as single sections or maximum intensity projections of the complete stack. All images for 1 experiment were taken at the same settings.

### 2.7 Live 2-photon imaging

Adult flies were fixed ventral side down to microscope slides using dental composite (3M Espe Sinfony Enamel Effect Material) and cured using a curing light (3TECH LED-1007 #104-0028). A small piece of cuticle was removed from the posterior side of the head (cuticular window) to reveal the fly brain. GFP fluorescence resulting from LanB2:GFP expression in Dilp2 neurons was imaged with a Leica TCS SP8 MP 2-photon microscope (Leica, Wetzlar, Germany).

### 2.8 Quantitative real-time PCR (qPCR)

Total RNA was isolated from adult fly heads using standard TRIzol (Invitrogen) protocols. RNA samples were treated with Turbo DNAse (Invitrogen) and converted to cDNA using oligo-dT primers and Superscript II reverse transcriptase (Invitrogen). Quantitative RT-PCR was performed using Power SYBR Green PCR Master Mix (ABI) in the Quant Studio 6 Flex system (Applied Biosystems, Thermo Fisher Scientific). Each sample was analyzed in duplicate, and values are the mean of 4 or 5 independent biological repeats. Relative quantities of transcripts were determined using the relative standard curve method normalized to Tub84B or Rp49. The following primer sequences (Eurofins, UK) were used in the analysis: Aβ_42_: 5′-CGATCCTTCTCCTGCTAACC-3′, 5′-CACCATCAAGCCAATAATCG-3′; BiP: 5′-TCTTGTACACACCAACGCAGG-3′, 5′-CAAGGAGCTGGGCACAGTGA-3′; Cdc14 (PP30015): 5′-GACTTTGGTCCGCTCAACATA-3′, 5′-CGGATTCATGGAGGTGTAGTGA-3’; Cg25C (PP1392): 5′-GATCGCGGGAGCGTTAGTC-3′, 5'-TCACGGAGTCCTGAATCGAAC-3; GFP: 5′-CTGTCCACACAATCTGCCCT-3′, 5′-TGCCATGTGTAATCCCAGCA-3; LanA (PP18459): 5′-TACGGAACACGATCATATCGACT-3′, 5′- CCTGGACAACGGAGGACTCT-3′; LanB1 (PP29286): 5′-CTCGCCGGAGAGATTCTGC-3′, 5′-TTGTACGGATCATGCTTGGTC-3′; Rp49: 5′-GACAATCTCCTTGCGCTTCT-3′, 5′-CCAGTCGGATCGATATGCTAA-3′; Tub84B: 5′-TGGGCCCGTCTGGACCACAA-3′, 5′-TCGCCGTCACCGGAGTCCAT-3′. Cdc14 (PP30015), LanA (PP18459), LanB1 (PP29286), and Cg25C (PP1392) primer sequences were obtained from the Fly Primer Bank (Hu et al., 2013) (flyrnai.org/flyprimerbank).

### 2.9 Quantification of total, soluble, and aggregated Aβ_42_

To extract total Aβ_42_, 5 fly heads were homogenized in 50 μL GnHCl extraction buffer (5 M Guanidinium HCl, 50 mM HEPES pH 7.3, protease inhibitor cocktail [Sigma, P8340] and 5 mM EDTA), centrifuged at 21,000 × g for 5 minutes at 4 °C, and cleared supernatant retained as the total fly Aβ_42_ sample. Alternatively, soluble and insoluble pools of Aβ_42_ were extracted using a protocol adapted from previously published methods (Burns et al., 2003, Sofola et al., 2010): 50 fly heads were homogenized in 50 μL tissue homogenization buffer (250 mM sucrose, 20 mM Tris base, 1 mM EDTA, 1 mM EGTA, protease inhibitor cocktail [Sigma]) then mixed further with 200 μL DEA buffer (0.4% DEA, 100 mM NaCl, and protease inhibitor cocktail). Samples were centrifuged at 135,000 × g for 1 hour at 4 °C (Beckman Optima Max centrifuge, TLA120.1 rotor), and supernatant was retained as the cytosolic, soluble Aβ_42_ fraction. Pellets were further resuspended in 400 μL ice-cold formic acid (70%) and sonicated for 2 × 30 seconds on ice. Samples were recentrifuged at 135,000 × g for 1 hour at 4 °C, then 210 μL of supernatant was diluted with 4 mL FA neutralization buffer (1 M Tris base, 0.5 M Na2 HPO4, 0.05% NaN3) and retained as the aggregated, formic acid-extractable Aβ_42_ fraction. Total, soluble, or aggregated Aβ_42_ content was measured using the ultrasensitive hAmyloid-β_42_ ELISA kit (Thermo Fisher, # KHB3544). Total and soluble Aβ_42_ samples were diluted 1:10, and aggregated Aβ_42_ samples were diluted 1:5 in sample/standard dilution buffer, and the ELISA was performed according to the manufacturers’ instructions. Protein extracts were quantified using the BCA Protein Assay Kit (Pierce), and the amount of Aβ_42_ in each sample was expressed as a ratio of the total protein content (pmol/g total protein).

### 2.10 Western blots

For protein extracts, fly heads (10–20 per biological sample) were homogenized in 1X NuPAGE LDS sample buffer (Thermo Fisher) with 200 mM DTT (Sigma) and boiled at 95 °C for 5 minutes. With the assumption that fly heads of the same sex contain comparable levels of total protein, we lysed 1 fly head per 10 μL sample buffer. Equal quantities of protein for each sample were then separated on 4%–12% NuPAGE Bis-Tris gels (Invitrogen) and transferred to a nitrocellulose membrane (GE Healthcare). For Aβ westerns, antigen retrieval was necessary, so membranes were boiled in 1X PBS for 5 minutes in a microwave. Membranes were blocked in 5% bovine serum albumin (BSA, Sigma) in Tris-buffered saline with 0.1% Tween-20 for 1 hour at room temperature, after which they were probed with primary antibodies overnight at 4 °C. The following primary antibodies were used: mouse anti-Aβ 6E10 (1:500; BioLegend #803001 previously #39320), rabbit anti-BiP (1:1000; Novus Biologicals #NBP1-06274), rabbit anti-Actin (1:5000; Abcam #ab1801), and mouse anti-Tubulin (1:2000, Sigma #T6199). Membranes were then probed with secondary anti-mouse HRP (1:10,000; Abcam #ab6789) or anti-rabbit HRP antibodies (1:10,000; Abcam #ab6721) for 1 hour at room temperature. Blots were developed using Luminata Crescendo (Millipore) and the ImageQuant LAS 4000 system. Densitometric analysis of blot images was carried out using Fiji software (Schindelin et al., 2012).

### 2.11 Single-cell transcriptomic data for the adult fly brain

Single-cell atlas images were obtained by gene name searches in *Scope* (Davie et al., 2018), the visualization and analysis tool from the Aerts Lab (http://scope.aertslab.org/).

### 2.12 Mice

P10 wild-type mice (C57/BL6J) were used for slice culture experiments. Animals were culled by humane schedule 1 methods and brain tissue was removed for the generation of organotypic slice cultures. All animal experiments conformed to national and institutional guidelines including the Animals (Scientific Procedures Act) 1986 (UK) and the Council Directive 2010/63EU of the European Parliament and the Council of September 22, 2010 on the protection of animals used for scientific purposes and had full Home Office ethical approval. Mice were bred in-house and group-housed on a 12 h/12 h light/dark cycle with *ad libitum* access to food and water.

### 2.13 Organotypic slice cultures

Organotypic cultures of the hippocampus and surrounding cortex were taken from humanely sacrificed P10 mouse pups of either sex according to previously described protocols (De Simoni and MY Yu, 2006; Durrant, 2020; Harwell and Coleman, 2016). Briefly, brains were rapidly removed and kept in dissection buffer (Earle’s Balanced Salt Solution + 25 mM HEPES + 1X Penicillin/Streptomycin) on ice. From this point, until plating, all equipment and tissue were kept ice-cold. Brains were bisected at the midline, then the cut sides glued (loctite super glue), face down onto a vibratome stage, and flooded with dissection media. Three hundred fifty micrometer sagittal slices (6 per brain) were taken using a Leica VT1200S vibratome; the hippocampus with surrounding cortex was dissected out using sterile syringe needles. The dissected slices were then transferred (using a sterile 3 mL plastic pipette—modified to widen the opening) to Falcon tubes full of ice-cold dissection medium and stored until plating. To plate, slices were transferred (3 slices from the same pup per dish, 2 dishes per pup [split randomly between control/test conditions]) onto sterile 0.4 µm pore membranes (Millipore #PICM0RG50) in 35 mm culture dishes (Nunc). Inserts were kept in 1 mL of maintenance medium (50% MEM with Glutamax-1 [Life Tech:42360-024], 25% heat-inactivated horse serum [Life Tech: 26050-070], 23% Earle’s balanced salt solution [Life Tech: 24010-043], 0.65% D-glucose [Sigma: G8270], 2% penicillin-streptomycin [Life Tech: 15140-122], and 6 units/mL nystatin [Sigma: N1638]) and cultures were maintained in incubators at 37 °C, 5% CO2 for 4 weeks. Two 100% medium exchanges occurred (5 hours after plating and 4 days *in vitro*) and a 50% media exchange occurred each week thereafter. At 14 days *in vitro*, LamB1 lentivirus (VectorBuilder: pLV[Exp]-EGFP:T2A:Puro-EF1A>mLamb1[NM_008482.3], Vector ID VB900104–1376qmp, https://en.vectorbuilder.com/vector/VB900104-1376qmp.html) or control lentivirus (VectorBuilder: EGFP control lentivirus [>10^8^ TU/mL, 100 μL, HBSS buffer], made from vector: VB160109–10005) was applied at a viral titer of 8.33 × 10^7^ per dish (diluted in the culture medium with a few drops added on top of the slice to ensure compete perfusion of the tissue). The culture medium was changed 24 hours later, and cultures were maintained for a further 14 days *in vitro* before fixation.

### 2.14 Immunofluorescence staining of organotypics

Slice cultures were fixed for 20 minutes in 4% paraformaldehyde in PBS. Slices were washed twice in PBS, blocked for 1 hour in blocking solution (PBS with 0.5% Triton X-100% and 3% goat serum) then incubated in 200 μL primary antibody diluted in blocking solution overnight at 4 °C with shaking. Slices were washed 3 times in PBS before being incubated (2 hours, RT in the dark) with secondary antibodies in the blocking solution. In the second of the final 3 PBS washes, slices were incubated with DAPI (Sigma, 1:10,000 in PBS). Images were captured using a Leica TCS8 confocal microscope (Leica, Wetzlar, Germany). Primary antibodies used: rat Lamb1 (1:250; Abcam #ab44941), rabbit Calnexin (1:250; Abcam #ab13504). Secondary antibodies used: goat anti-rabbit Alexa Fluor 594 (1:400; Invitrogen #A11037) and goat anti-rat Alexa Fluor 647 (1:400; Invitrogen #A21247).

### 2.15 Statistics

Data were grouped for each genotype, and the mean (±SEM) was calculated. Log-rank, Cox proportional hazard, analysis of variances, Kruskal-Wallis, and the appropriate post-hoc analyses were performed. If the model did not meet the assumptions (assessed by plotting model residuals against predictors, normality of residuals was checked with a QQ-plot, and homogeneity of variance checked by plotting residuals against fitted values), data were transformed using the method that was best transformed each individual model to fit the assumptions, for example, log transformation. Statistical analyses were performed in Excel (Microsoft) or Prism (GraphPad, La Jolla, CA), except for Cox proportional hazards, which were performed in R Studio (R Core Team). A statistical difference of *p* < 0.05 was regarded as significant.

## 3 Results

### 3.1 Overexpression of the laminin β-chain LanB1 rescues the toxic effect of neuronal Aβ42 expression

To test if modulation of ECM components could alter the toxicity of Aβ_42_ *in vivo*, we took advantage of the Aβ_42_-shortened lifespan of *Drosophila* as a read-out. We used a drug-inducible *Drosophila* AD model that expressed the highly aggregative Arctic Aβ_42_ (Aβ^Arc^) (Crowther et al., 2005), which when expressed in the neurons of adult flies shortens lifespan and induces behavioral defects and neurodegeneration (Casas-Tinto et al., 2011, Sofola et al., 2010). The Aβ sequence contains a signal peptide sequence from the *necrotic* gene (Crowther et al., 2005) to target it to the secretory pathway, resulting in Aβ that is secreted into the extracellular milieu. The pan-neuronal ElavGS driver can be induced by RU486 (“RU,” a steroid drug inducer) to switch on gene expression. We generated ElavGS; Aβ^Arc^ recombinant flies and used these to screen ECM-related genes by driving co-expression in neurons during adulthood. We observed a pronounced rescue of lifespan with co-expression of Aβ and the β subunit of Laminin (LanB1), using the EP-600 line with a P-element inserted 548 bp upstream of the *LanB1* gene (Urbano et al., 2009), compared to the Aβ^Arc^-alone controls (Supplementary Fig. 1A). We examined further laminin transgenic lines and found that 2 independent LanB2 RNAi lines also significantly ameliorated Aβ^Arc^ toxicity, but not to the same extent as LanB1 co-expression (Supplementary Fig. 1B). As LanB1 co-expression induced the most consistent and pronounced rescue of Aβ^Arc^ toxicity, we took this line forward for further study.

Previous studies have shown that, during *Drosophila* development, laminin is predominantly produced by the fat body, hemocytes, and glia (Bunt et al., 2010, Pastor-Pareja and Xu, 2011, Petley-Ragan et al., 2016). Information about laminin expression in the adult fly brain is less abundant. However, by examining gene expression in the adult fly brain using *SCope* (Davie et al., 2018), a single-cell transcriptome atlas of the adult *Drosophila* brain, we found that expression of laminin subunits appeared to be specific to hemocytes and a subset of glial cells, while endogenous expression in neurons was low (Supplementary Fig. 2A and B), indicating that neuronal expression of these subunits is mainly ectopic.

To establish the robustness of the rescue of Aβ toxicity and to eliminate potential confounding effects of genetic background, we tested further fly lines in the standardized Dahomey (*w*^Dah^) genetic background. The UAS-LanB1^EP-600^ mutant was backcrossed, and the lifespan rescue experiment was repeated, further including the genetically identical, uninduced controls. Co-expression of LanB1 and Aβ^Arc^ in females resulted in a significant rescue of the short-lived phenotype (Fig. 1A, repeat experiments, Fig. 1B and Supplementary Fig. 1C). The rescue was also significant in male flies (Fig. 1C). We next assessed the ability of LanB1 co-expression to rescue the shortened lifespan in a different fly AD model, in which 2 copies of wild-type, tandem Aβ_42_ (Aβ^X2^) (Casas-Tinto et al., 2011) with an export sequence from the *Argos* gene are expressed in adult neurons. Co-expression of an RFP-tagged LanB1 significantly rescued the short lifespan with Aβ^X2^ induction (Fig. 1D and Supplementary Fig. 1D). To assess if these neuronal effects were specific to the ElavGS driver, we used a second, independent, pan-neuronal GeneSwitch driver, NsybGS. LanB1 significantly rescued the shortened lifespan with Aβ^Arc^ induction in NsybGS flies (Fig. 1E).

**Fig. 2:**
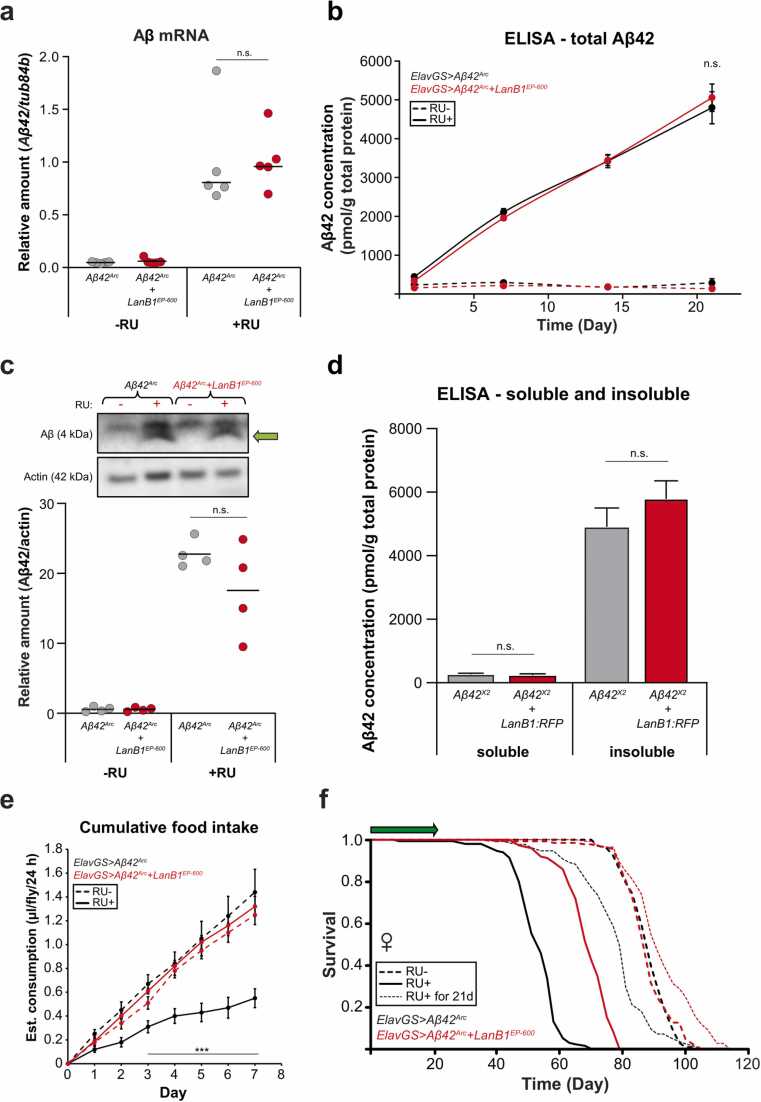
**LanB1 did not affect Aβ RNA or protein levels**. (A) Aβ mRNA was significantly upregulated upon RU induction (*p* < 0.0001; 1-way analysis of variances (ANOVA) with Tukey’s post-hoc test) and was not affected by co-expression of LanB1. Data indicate the mean (n = 5 biological replicates per condition). (B) Total Aβ_42_ protein levels increased significantly over time with RU induction compared to uninduced controls (*p* < 0.0001, 2-way ANOVA with Tukey’s post-hoc test), and were unaffected by LanB1 co-expression. Data are shown as mean ± SEM (n = 4 biological replicates per time point). (C) Western blot with quantification of soluble Aβ. The green arrow indicates the distinctive crescent shape of Aβ used for quantification. Soluble Aβ levels increased in induced conditions and were unaffected by LanB1 co-expression. Data indicate the mean (n = 4 biological replicates per condition). (D) After 3 weeks of induction, neither soluble nor insoluble Aβ levels were affected by LanB1 co-expression. Data are shown as mean ± SEM (n = 7 biological replicates per condition). (E) After 3 weeks of RU, induced Aβ^Arc^ flies consumed a significantly smaller amount of food over 7 days compared to uninduced controls (*p* < 0.0001, 2-way ANOVA with Tukey’s post-hoc test). The amount of food ingested by flies with a history of LanB1 and Aβ^Arc^ induction was not significantly different from uninduced controls. Food consumption was measured for 10 flies per condition every 24 hours. Data are shown as mean ± SEM. (F) Expression of Aβ for the first 3 weeks of adulthood significantly reduced lifespan compared to uninduced controls (*p* = 4.07 × 10^−12^; log-rank test). Three-week induction of Aβ and LanB1 together resulted in a small but significant lifespan extension compared to uninduced controls (*p* = 5.04 × 10^−06^; log-rank test). Abbreviations: Aβ, amyloid β; ElavGS, Elav-GeneSwitch; LanB1, Laminin B1.

To assess the effects of LanB1 on neuromuscular performance in AD flies, we measured their climbing behavior. Expression of Aβ^Arc^ in adult neurons led to a significant decline in climbing ability compared to uninduced controls (Fig. 1F), and LanB1 co-expression significantly rescued the deficit. We also examined the feeding behavior of the flies, using the CAFE assay (Ja et al., 2007). Flies were induced/uninduced for 3 weeks, and then feeding behavior was measured with all flies eating food without RU. Aβ^Arc^ flies ingested a significantly smaller amount of food over 7 days compared to uninduced controls, while LanB1 co-expression completely rescued feeding behavior to uninduced levels (Fig. 2E).

Expression of Aβ can also have toxic effects during development. For instance, expression of Aβ^X2^ using the GMR-GAL4 driver causes degeneration of the developing eye, resulting in a smaller, glassier appearance compared to the large eye and highly ordered ommatidial lattice in control flies. LanB1 co-expression led to a rescue of both the size and organization of the eye (Fig. 1G). LanB1 could therefore rescue multiple toxic effects of Aβ expression.

### 3.2 Aβ is not toxic in glia in adult flies

We also induced adult-onset expression of Aβ in the other major cell type in the fly brain, glia, and examined whether LanB1 co-expression could rescue any deleterious phenotypes. Pan-glial expression of Aβ^Arc^ did not reduce lifespan compared to uninduced controls, nor did Aβ and LanB1 co-expression (Supplementary Fig. 1E). This result is in line with the recent finding that Aβ produced by glia was less toxic despite having a much higher brain load than with neuronal expression (Jonson et al., 2018, Marcora et al., 2017).

### 3.3 LanB1 rescue appears specific to Aβ

We tested the effect of LanB1 co-expression in fly neurodegeneration models with different toxic peptides. Neuronal expression of long polyglutamine tracts (polyQ) such as those found in Huntington’s disease is toxic and results in shortened lifespan (Marsh et al., 2000). Similarly, adult-onset neuronal expression of the expanded GGGGCC repeat ((G_4_C_2_)_36_) in C9orf72 (the most common genetic cause of frontotemporal dementia and amyotrophic lateral sclerosis) is highly toxic and reduces lifespan (Mizielinska et al., 2014). LanB1 and polyQ co-expression resulted in a small but significant reduction in lifespan compared to polyQ-alone controls (Supplementary Fig. 1F), and LanB1 and (G_4_C_2_)_36_ co-expression resulted in a significant reduction in lifespan compared to (G_4_C_2_)_36_-alone controls (Supplementary Fig. 1G). These results suggest that the beneficial effects of LanB1 may be specific to Aβ toxicity, since LanB1 did not rescue these other proteotoxic stressors.

### 3.4 *LanB1*, and not *cdc14*, an adjacent gene in the opposite genomic orientation, is responsible for the rescue of Aβ toxicity

The LanB1^EP-600^ P-element (P{GSV1}) contains UAS sequences at both ends oriented outward (Supplementary Fig. 3A) (Toba et al., 1999) and could potentially drive expression of *cdc14,* an adjacent gene in the opposite genomic orientation, previously reported to be involved in stress-resistance and lipid metabolism (Neitzel et al., 2018). To determine if the expression of *cdc14* was affected, we performed qPCR on the heads of ElavGS>Aβ^Arc^ flies with and without RU induction and examined the expression of *LanB1* and *cdc14*. We confirmed ∼10-fold increase in *LanB1* gene expression when induced in both UAS-LanB1^EP-600^ and UAS-LanB1:RFP flies (Supplementary Fig. 3B), and no change in UAS-cdc14^EY10303^ flies. As predicted, *cdc14* expression was significantly upregulated in both UAS-cdc14^EY10303^ and UAS-LanB1^EP-600^ flies, but not in UAS-LanB1:RFP transgenic flies (Supplementary Fig. 3C).

To determine if LanB1, cdc14, or both were protective against Aβ toxicity, we expressed them singly and examined the effect on eye phenotypes. Upregulation of cdc14 alone during development, using UAS-cdc14^EY10303^, did not rescue the toxic effects of Aβ^X2^, while UAS-LanB1:RFP rescued both the size and organization of the eye to the same extent as in UAS-LanB1^EP-600^ flies (Supplementary Fig. 3D and quantified in Supplementary Fig. 3E). We also tested whether cdc14 alone could rescue the short lifespan of ElavGS>Aβ^Arc^ flies. Upregulation of cdc14 caused a small rescue of Aβ toxicity compared to induced ElavGS>Aβ^Arc^ controls, although a similar small rescue was observed with cytoplasmic GFP overexpression (Supplementary Fig. 3F). LanB1 upregulation, via either UAS-LanB1^EP-600^ or UAS-LanB1:RFP, caused an equivalently pronounced rescue of Aβ toxicity. Thus, LanB1 and not *cdc14* were responsible for the rescue of Aβ toxicity in UAS-LanB1^EP-600^ flies.

### 3.5 Co-expression of LanB1 does not lower levels of Aβ

A potential explanation for the rescue of Aβ toxicity by LanB1 is that less Aβ transcript was generated due to GAL4 titration by the second UAS transgene. We therefore measured Aβ mRNA and protein levels. Aβ was increased >5-fold when induced in either the presence or absence of the LanB1 transgene (Fig. 2A), ruling out GAL4 titration as a factor in the rescue of Aβ toxicity. We then examined the dynamics of Aβ peptide accumulation over a 3-week period by ELISA and found no difference between flies expressing Aβ alone and those co-expressing LanB1 (Fig. 2B). We then measured the levels of soluble and insoluble Aβ by western blot (Fig. 2C) and ELISA (Fig. 2D). There was no significant difference in the levels of soluble or insoluble Aβ between flies expressing Aβ alone and those co-expressing LanB1. Thus, there was no difference in Aβ expression at either the RNA or protein level between flies expressing Aβ alone and those co-expressing LanB1, or was the solubility state of Aβ affected, indicating that LanB1 rescued Aβ toxicity rather than Aβ expression.

### 3.6 Rescue of Aβ toxicity by acute LanB1 overexpression implicates soluble Aβ as the main driver of toxicity

Experiments where expression of Aβ is temporarily induced have shown that high levels of insoluble Aβ persist in the brain long after induction has ceased, while soluble Aβ appears to be rapidly cleared (Rogers et al., 2012). Induction of Aβ in the first weeks after eclosion results in reduced climbing ability and survival (Rogers et al., 2012). It remains unclear if the detrimental effects of Aβ on climbing and lifespan in later life are caused by the long-lasting toxic effects of soluble Aβ during the early-life induction period, from the insoluble Aβ that persists in the aging brain, or both. We found that 21 days of Aβ induction resulted in a significantly reduced lifespan compared to uninduced controls (Fig. 2F), although the reduction was not as great as with chronic Aβ induction. Co-expression of both Aβ and LanB1 for 21 days, in contrast, resulted in a lifespan extension compared to uninduced controls (Fig. 2F). These results indicate that the rescue occurred during the early induction period, and that, therefore, soluble Aβ during the induction phase, rather than effects of accumulated insoluble Aβ, may have been responsible for the later effect on lifespan.

### 3.7 Laminin accumulates in the ER

**Fig. 3:**
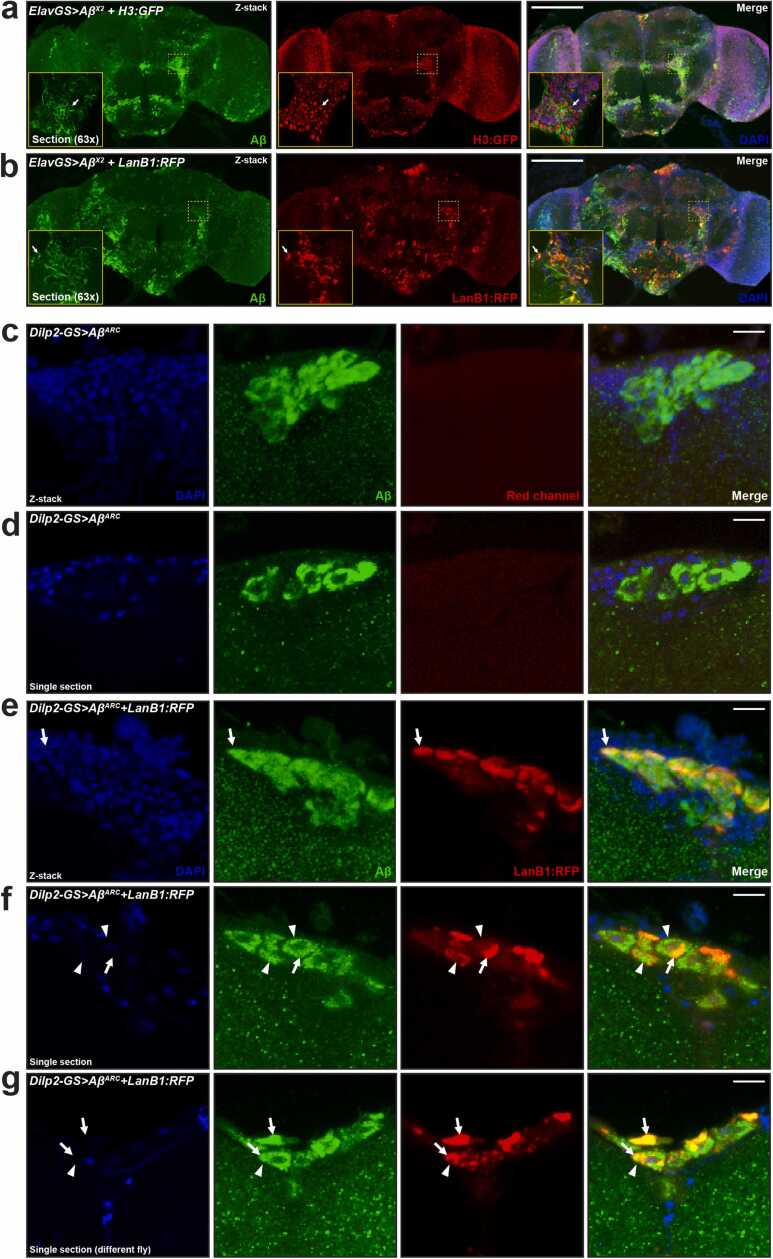
**Intracellular accumulation of Aβ and LanB1**. (A) Aβ accumulated intracellularly and did not colocalize with the nuclear marker histone H3:GFP. The arrow indicates the non-nuclear expression of Aβ. (B) Similarly, LanB1 accumulated intracellularly, not in the nucleus, and partially overlapped with Aβ expression. The arrow indicates the colocalization of Aβ and LanB1. (A and B) Representative confocal fluorescence z projections taken at 20× magnification of whole brains from 21-day-old female flies stained with Aβ (6E10-green) and DAPI (blue). The yellow box inset shows a single section of the same brain taken at 63× magnification. Endogenous fluorescence (i.e., without staining) of H3:GFP and LanB1:RFP is shown. H3:GFP has been false-colored red to aid comparison. (C) Z-projection and (D) single section showing intracellular accumulation of Aβ in Dilp2 neurons. (E) Z-projection and (F and G) single sections showing intracellular accumulation of Aβ and LanB1 in Dilp2 neurons. LanB1 expression was not uniformly distributed through the cytoplasm of these cell bodies and appeared to accumulate in discrete intracellular compartments. Arrows highlight the colocalization of Aβ and LanB1, while arrowheads highlight areas with no overlap, often in the same cell. (C-G) Representative confocal images taken at 63x magnification of Dilp2 neurons from 21-day-old female flies stained with Aβ (6E10-green) and DAPI (blue). Endogenous fluorescence (i.e., without staining) of LanB1:RFP is shown. Genotypes: (A) *ElavGS>Aβ*^*X2*^*+ H3:GFP*; (B) *ElavGS*>*Aβ*^*X2*^*+ LanB1:RFP*; (C and D) *Dilp2-GS>Aβ^Arc^*; (E-G) *Dilp2-GS*>*Aβ^Arc^* + *LanB1:RFP*. Scale bar, 100 µm for (A and B), 10 µm for (C-G). Abbreviations: Aβ, amyloid β; Dilp2, *Drosophila* insulin-like peptide 2; ElavGS, Elav-GeneSwitch; LanB1, Laminin B1.

The laminin heterotrimer is a canonical ECM protein and is secreted into the extracellular space (Hellewell and Adams, 2016). Overexpression of monomeric RFP-tagged LanB1, however, leads to intracellular retention (Liu et al., 2017). To determine the cellular localization of LanB1, adult brains expressing Aβ and LanB1:RFP were dissected, stained, and imaged. In agreement with previous studies (Crowther et al., 2005, Jonson et al., 2018, Ray et al., 2017), we found that overexpression of the Aβ^Arc^ peptide resulted in aggregation of Aβ in neurons (Fig. 3A). Aβ did not overlap with the nuclear protein histone H3. LanB1 accumulated intracellularly and partially overlapped with Aβ (Fig. 3B).

Upon closer inspection, LanB1 appeared to accumulate in discrete intracellular puncta or compartments. To verify the intracellular expression of LanB1 in neurons, we used the inducible Dilp2-GS driver, which drives expression in the Dilp2 neurons. *Drosophila* insulin-like peptide 2 (Dilp2) neurons consist of 10–14 insulin-like peptide-producing cells located in the dorsal brain *pars intercerebralis* that project their axons ventrally (Nässel et al., 2013). Driving LanB1 expression in these neurons allowed us to observe whether or not LanB1 was secreted into the extracellular space and/or accumulated intracellularly. We verified that Aβ and LanB1 were induced in these neurons only when the flies were fed RU486 (Supplementary Fig. 4) and that Aβ in Dilp2 neurons accumulated predominantly in the soma (Fig. 3C and D). Similar to Aβ, LanB1 accumulated intraneuronally but did not completely overlap with Aβ (Fig. 3E–G). LanB1 was not uniformly distributed through the cytoplasm of these cell bodies and appeared to accumulate in discrete intracellular compartments.

**Fig. 4:**
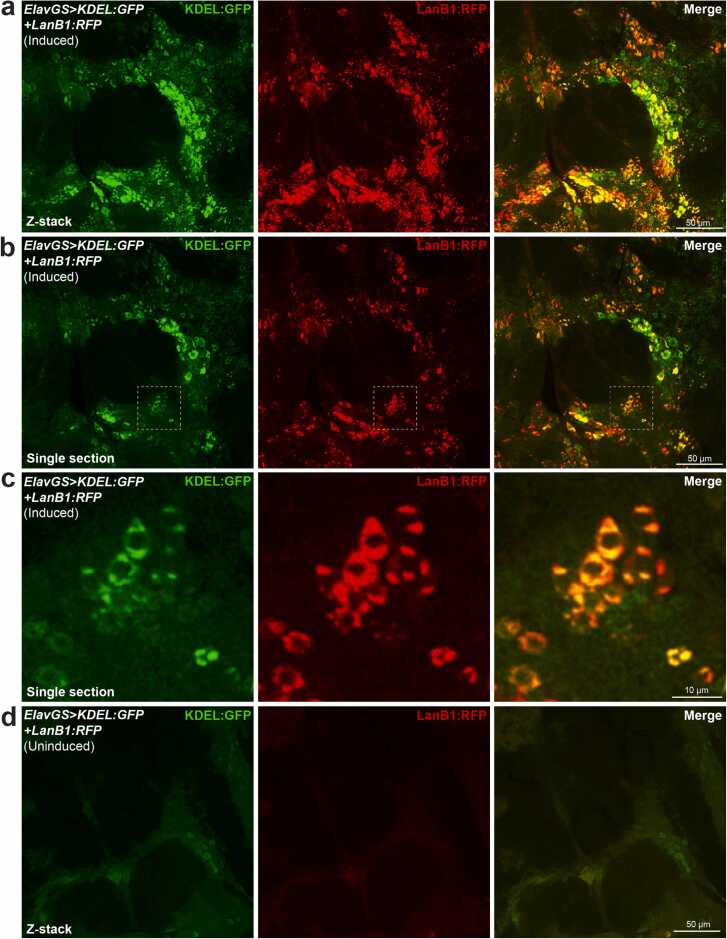
**LanB1 accumulates in the ER**. (A) Z-projection and (B) single section showing neuronal expression of LanB1 with the ER marker, KDEL:GFP, using the ElavGS driver. (C) Magnified view of the dashed yellow box inset in (B). LanB1 colocalizes with KDEL:GFP. (D) Without RU486 induction, there was very little induction of LanB1 and/or KDEL:GFP expression. Representative confocal fluorescence images were taken at 63× magnification from 7-day-old female flies. Images show the antennal lobe and surrounding brain regions. Endogenous fluorescence (i.e., without staining) of KDEL:GFP and LanB1:RFP is shown. Genotype: *ElavGS*>*KDEL:GFP + LanB1:RFP*. Scale bar, 50 µm for (A, B, and D), 10 µm for (C). Abbreviations: Dilp2, *Drosophila* insulin-like peptide 2; ElavGS, Elav-GeneSwitch; LanB1, Laminin B1.

As laminin β and γ subunits cannot be secreted from the ER without first forming a heterotrimer (Chen et al., 2013, Miner and Yurchenco, 2004), overexpressing LanB1 could have led to its accumulation in the ER (Petley-Ragan et al., 2016). Indeed, intracellular accumulation of LanB1 protein has been previously reported for expression of the LanB1:RFP transgene in the fat body (Liu et al., 2017). Similarly, the expression of the LanB1^EP-600^ element also led to intracellular laminin accumulation (de Celis and Molnar, 2010, Petley-Ragan et al., 2016). To test if the intracellular accumulation of LanB1 was in the ER, we co-expressed LanB1:RFP and a GFP-tagged ER marker, KDEL:GFP, in adult neurons. We found a strong overlap of KDEL:GFP and LanB1:RFP expression, indicating that LanB1 accumulated in the ER (Fig. 4). We also found intracellular accumulation, probably in the ER, of LanB2:GFP in Dilp2 neurons when we examined these flies by live 2-photon imaging (Supplementary Fig. 6).

To determine whether intracellular Aβ aggregates were localized to the ER, we co-expressed Aβ and KDEL:GFP in adult Dilp2 neurons. Aβ did not colocalize with KDEL:GFP, indicating that Aβ did not accumulate in the ER (Supplementary Fig. 7). These results are in line with previous studies in the adult fly brains that demonstrated that Aβ mainly accumulates in lysosomes (Ling et al., 2009, Ling and Salvaterra, 2010), and it also does so in mice expressing intraneuronal Aβ (Abramowski et al., 2012).

### 3.8 LanB1 does not reduce secretion of Aβ from neurons

**Fig. 5:**
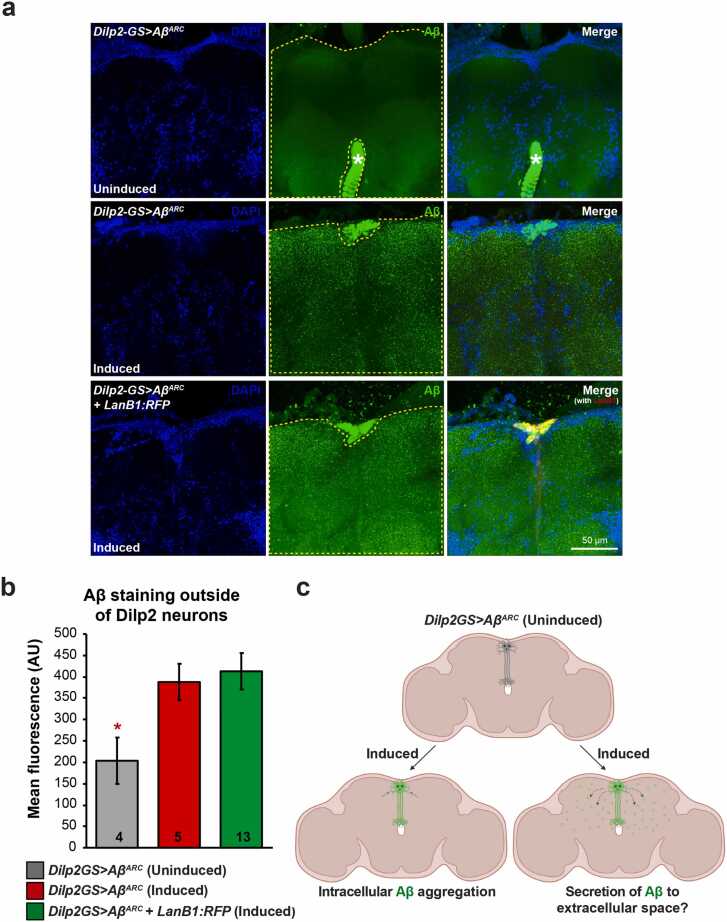
**Quantification of Aβ secretion from Dilp2 neurons**. (A) Aβ expression in Dilp2 neurons was highest in the cell body area, but there was also diffuse, punctate staining of Aβ outside the cell bodies. LanB1 co-expression had no effect on Aβ fluorescence outside Dilp2 neurons. Without RU induction, there was no Aβ found in Dilp2 neurons or the surrounding area. Representative confocal fluorescence z projections taken at 63× magnification of whole brains from 21-day-old female flies stained with Aβ (6E10-green) and DAPI (blue). The dashed yellow area indicates the area of fluorescence measurement. White asterisk in the top row of images indicates strong staining of esophageal muscle. Endogenous fluorescence (i.e., without staining) of LanB1:RFP is also shown. (B) Quantification of Aβ fluorescence outside Dilp2 neurons. There was a significant increase in diffuse, punctate Aβ staining outside Dilp2 neurons when induced (*p* = 0.048; 1-way analysis of variances). LanB1 co-expression had no effect on Aβ staining. Biological replicate numbers for each condition are labeled within the bars. (C) Diagram of the proposed secretion of Aβ from Dilp2 neurons into the extracellular space. Created with BioRender.com. Genotypes: *Dilp2-GS*>*Aβ^Arc^*; and *Dilp2-GS*>*Aβ^Arc^ + LanB1:RFP*. Scale bar, 50 µm. Abbreviations: Aβ, amyloid β; Dilp2, *Drosophila* insulin-like peptide 2; LanB1, Laminin B1.

Since Aβ protein levels, both soluble and insoluble, were not reduced in LanB1 co-expressing flies, we hypothesized that the intracellular accumulation of LanB1 might have prevented the normal secretion of the toxic Aβ peptide into the extracellular space, despite the presence of a signal peptide. We used the Dilp2-GS line to drive expression during the first 3 weeks of adulthood in Dilp2 neurons and then measured Aβ fluorescence in the area outside of Dilp2-expressing cells. We found that, when induced, Aβ was highly expressed in the cell bodies of Dilp2 neurons and also displayed a diffuse punctate pattern of staining, compared to very little staining in uninduced flies (Fig. 5A). When quantified, we found a significant increase in Aβ fluorescence in induced flies compared to uninduced controls, indicating that Aβ is secreted into the extracellular environment (Fig. 5B). LanB1 co-expression had no effect on the secretion of Aβ. In summary, Aβ aggregated strongly in the cell bodies of Dilp2 neurons and was also secreted into the extracellular milieu (Fig. 5C). Therefore, the beneficial effects of LanB1 co-expression were not due to the prevention of Aβ secretion.

### 3.9 Increased expression of a collagen IV subunit also rescues Aβ toxicity in adult neurons

**Fig. 6:**
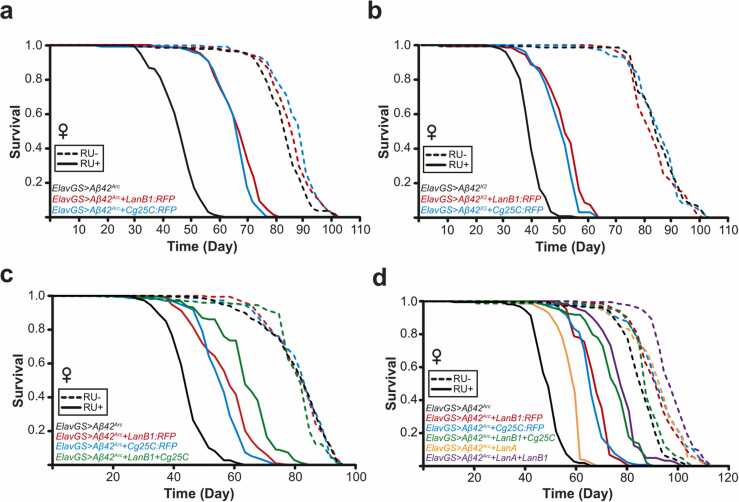
**Enhanced rescue of Aβ toxicity with combined laminin/collagen IV subunit overexpression**. (A) LanB1 significantly rescued Aβ toxicity (*p* = 2.85 × 10^−62^; log rank). Cg25C significantly rescued Aβ toxicity (*p* = 3.42 × 10^−63^; log rank). There were small but significant extensions of lifespan in the uninduced controls (LanB1, *p* = 0.036; Cg25C, *p* = 7.51 × 10^−06^; log rank vs. *ElavGS*>*Aβ^Arc^* alone). (B) LanB1 significantly rescued Aβ toxicity (*p* = 8.04 × 10^−40^; log rank). Cg25C significantly rescued Aβ toxicity (*p* = 1.25 × 10^−35^; log rank). (C) LanB1 significantly rescued Aβ toxicity (*p* = 3.77 × 10^−33^; log rank). Cg25C significantly rescued Aβ toxicity (*p* = 2.24 × 10^−30^; log rank). LanB1 + Cg25C had a partially additive effect on the rescue of Aβ^Arc^ toxicity. Cox proportional hazard analysis showed a significant interaction between LanB1 and Cg25C (*p* < 0.001). (D) LanB1, Cg25C, and LanA rescued Aβ toxicity (LanB1, *p* = 3.34 × 10^−65^; Cg25C, *p* = 2.11 × 10^−55^; LanA, *p* = 1.10 × 10^−32^; log rank vs. *ElavGS*>*Aβ*^Arc^ alone). LanB1 + Cg25C and LanB1 + LanA had a partially additive effect on the rescue of Aβ^Arc^ toxicity. Cox proportional hazard analysis showed a significant interaction with LanB1 for Cg25C (*p* < 0.001) and LanA (*p* = 0.022). There was a significant extension of lifespan in the uninduced controls (LanB1, *p* = 6.45 × 10^−10^; Cg25C, *p* = 1.57 × 10^−11^; LanA, *p* = 1.40 × 10^−10^; LanA + LanB1, *p* = 1.66 × 10^−39^; LanB1 + Cg25C, *p* = 0.026; log rank vs. *ElavGS*>*Aβ^Arc^* alone). Dashed lines represent uninduced “RU−” controls, and solid lines represent induced “RU+” conditions. For all lifespan experiments, n = 150 flies per condition. Abbreviations: Aβ, amyloid β; ElavGS, Elav-GeneSwitch; LanB1, Laminin B1.

The trimerization of laminins takes place in the ER, and all 3 subunits are required for secretion to occur (Yurchenco et al., 1997). We hypothesized that overexpressing protein subunits of similar, large, obligate heterotrimers that assemble in the ER may lead to amelioration of Aβ toxicity. Collagen IV, the main component of basement membranes, is another obligate heterotrimeric protein formed by 3 α chains (2 α chains of collagen at 25C [Cg25C] and 1 α chain Viking [Vkg]) and is present in all metazoans (Fidler et al., 2017, Natzle et al., 1982). Similar to laminins, expression of collagen IV subunits in the adult fly brain was predominantly in hemocytes and a subset of glial cells, while endogenous expression in neurons was low (Supplementary Fig. 2C), indicating that pan-neuronal expression of these subunits by ElavGS or NsybGS is ectopic. The RFP-tagged Cg25C transgenic overexpression line (*UAS-Cg25C:RFP*) rescued the short lifespan from induction of Aβ^Arc^ (Fig. 6A) and Aβ^X2^ (Fig. 6B), and to the same degree as LanB1, potentially indicating a similar mechanism of action.

We next examined if the rescue of degeneration in the fly eye due to Aβ toxicity (Fig. 1F) was specific to LanB1. Co-overexpression of mCD8:GFP or mCD8:RFP alone, as controls for GFP/RFP transgene overexpression, had no significant effect on Aβ toxicity (Supplementary Fig. 5A and quantified in Supplementary Fig. 5B). First, we assessed the effect of modulation of other laminin/collagen subunits on the rough eye phenotype. We confirmed that LanB1 co-expression (either using *UAS-LanB1*^*EP-600*^ or *UAS-LanB1:RFP*) significantly rescued Aβ toxicity. Additionally, we found that the co-expression of laminin subunits, LanA (*UAS-LanA*^*EY02207*^) and LanB2 (*UAS-LanB2:GFP*), and the collagen IV subunit Cg25C (*UAS-Cg25C:RFP*) separately all rescued Aβ toxicity (Supplementary Fig. 5). Knockdown of laminin α-chain subunits, LanA and wb, using TRiP RNAi transgenics had no effect on toxicity, while knockdown of γ-chain LanB2 led to a small but significant rescue (Supplementary Fig. 5). Next, we generated recombinant transgenic lines for co-expression of LanB1, that is, “LanB1^EP-600^ + Cg25C:RFP,” “LanB1^EP-600^ + LanA^OE^,” “LanB1^EP-600^ + LanB2^TRiP^,” and “LanB1^EP-600^ + wb^TRiP^.” Although Cg25C and LanA could individually rescue eye size, modulation of these other subunits had no effect on the ability of LanB1 to protect against Aβ toxicity, indicating that LanB1 was the primary cause of the toxicity rescue in the context of the developing eye (Supplementary Fig. 5).

### 3.10 Enhanced rescue of Aβ toxicity with combination of laminin and collagen subunits

As both LanB1 and Cg25C individually rescued Aβ toxicity to a similar extent (Fig. 6A and B), and the combination of both did not further rescue Aβ toxicity in the developing eye (Supplementary Fig. 5), we hypothesized that these proteins may have been epistatic, that is, acting via the same mechanism. We therefore examined the effect of the combination of LanB1 and Cg25C in adult neurons on lifespan. We found that LanB1 and Cg25C together resulted in a greater rescue of Aβ toxicity compared to individual co-expression (Fig. 6C). Cox proportional hazard analysis showed a significant interaction between LanB1 and Cg25C, indicating that, although the enhanced rescue was partially additive, it was also acting via a shared pathway.

Laminin A (LanA) is one of two laminin α-chains in *Drosophila*, the other being *wing blister* (*wb*), which form heterotrimers with LanB1 (β-chain) and LanB2 (γ-chain) (Urbano et al., 2009). We found that overexpression of LanA alone could significantly rescue Aβ toxicity, but not to the same degree as LanB1 or Cg25C alone (Fig. 6D and Supplementary Fig. 8A). This is potentially due to LanA being produced at lower levels compared to LanB1 or Cg25C. We confirmed this via qPCR and found that LanA was upregulated by ∼7-fold whereas LanB1 and Cg25C had a >50-fold increase compared to controls (Supplementary Fig. 8C). We generated double transgenic flies with both LanA and LanB1, which resulted in an even greater rescue of toxicity, indicating an additive effect. However, Cox proportional hazard analysis showed a significant interaction between LanB1 and LanA, indicating that the enhanced rescue is partly acting via a shared pathway. LanA also rescued Aβ toxicity in the developing fly eye when compared to Aβ^X2^ alone, though not to the same extent as LanB1 (Supplementary Fig. 5). These results indicate that LanA can rescue Aβ toxicity, but to a lesser degree than LanB1, and this can be explained by its lower overexpression levels. Overall, the collagen IV subunit Cg25C and the laminin α chain LanA led to enhanced rescue of Aβ toxicity in combination with the laminin β chain LanB1. These effects were epistatic, suggesting an overlapping molecular pathway.

### 3.11 LanB1 rescue of Aβ toxicity is independent of the BiP/Xbp1 ER stress response pathway

**Fig. 7:**
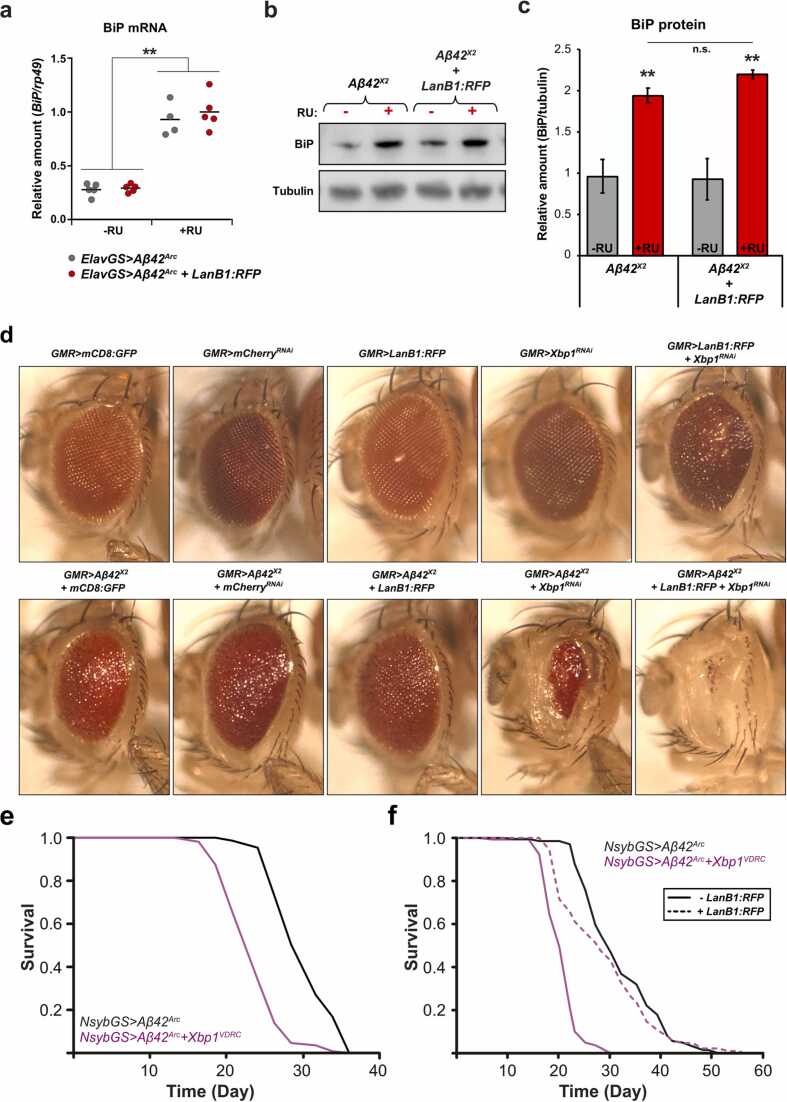
**LanB1 rescue of Aβ toxicity is independent of the BiP/Xbp1 ER stress response pathway**. (A) *BiP* mRNA was significantly upregulated upon Aβ induction (*p* < 0.01; 1-way analysis of variances with Tukey’s post-hoc test), and this was not changed by the overexpression of LanB1:RFP. Data are shown indicating the mean (n = 4–5 biological replicates per condition). (B) Western blot of BiP protein levels, and quantification in (C) confirm that BiP protein is also significantly upregulated with Aβ induction, and this is not changed by LanB1:RFP overexpression (*p* < 0.01; 1-way analysis of variances with Tukey’s post-hoc test). Data are shown indicating the mean (n = 3–4 biological replicates per condition). (D) Co-expression of Aβ^X2^ with Xbp1^VDRC^ in the developing eye. Knockdown of Xbp1 significantly exacerbated Aβ toxicity, and these flies exhibited very small and depigmented eyes. LanB1:RFP expression further enhanced the combined toxicity of Aβ and Xbp1^VDRC^. The combination of LanB1:RFP and Xbp1^VDRC^ without Aβ expression also resulted in a rough eye phenotype. (E) Knockdown of Xbp1 exacerbated Aβ toxicity and significantly shortened lifespan compared to controls (*p* = 3.03 × 10^−30^; log rank). (F) Knockdown of Xbp1 exacerbated Aβ toxicity and significantly shortened lifespan compared to controls (*p* = 1.68 × 10^−44^; log rank). Overexpression of LanB1 rescued the shorter lifespan of Xbp1^VDRC^ (Aβ+LanB1:RFP+Xbp1^VDRC^ vs. Aβ+Xbp1^VDRC^, *p* = 9.13 × 10^−22^; log rank). For display purposes, the control micrograph in (D) is the same as that in Supplementary Fig. 5. For all lifespan experiments, n = 150 flies per condition. “VDRC” indicates an RNAi transgene. Genotypes: (A) *ElavGS*>*Aβ*^Arc^; *ElavGS*>*Aβ*^Arc^*+ LanB1:RFP*; (B and C) *NsybGS*>*Aβ*^*Arc*^; *NsybGS*>*Aβ*^*Arc*^*+ LanB1:RFP*. Abbreviations: Aβ, amyloid β; ElavGS, Elav-GeneSwitch; LanB1, Laminin B1.

Aβ induces ER stress markers, including the ER chaperone BiP, and leads to increased alternative splicing of Xbp1 (Casas-Tinto et al., 2011, Marcora et al., 2017, Niccoli et al., 2016). Increased BiP can exacerbate Aβ toxicity while reduction of BiP has been shown to have beneficial effects (Niccoli et al., 2016, Sekiya et al., 2017). To assess if BiP reduction was behind the LanB1 rescue of Aβ toxicity, we examined BiP mRNA and protein levels. After 3 weeks of Aβ induction, BiP was significantly upregulated at both the mRNA (Fig. 7A) and protein level (Fig. 7B and C) in fly heads. Co-expression of LanB1 with Aβ had no effect on BiP mRNA or protein levels. Therefore, LanB1 rescued Aβ toxicity independently of the ER stress regulator, BiP.

An increase in active spliced Xbp1, a marker of IRE1α activation, can ameliorate Aβ toxicity, while a decrease exacerbates Aβ toxicity (Casas-Tinto et al., 2011, Marcora et al., 2017). To assess whether the LanB1 rescue was acting via Xbp1, we examined the effect of Xbp1 knockdown on Aβ toxicity. We used the Aβ-induced rough eye phenotype, and Aβ-induced short lifespan as read-outs. While there was no effect of knockdown of Xbp1 alone on the developing eye, Xbp1^RNAi^ significantly exacerbated Aβ toxicity in the developing eye (Fig. 7D). The combination of Xbp1^RNAi^ and Aβ also led to a significantly shortened lifespan compared to Aβ alone (Fig. 7E and F). There was also no effect of LanB1 expression alone on eye development, but when in combination with Xbp1^RNAi^ resulted in a rough eye, indicating the toxic potential of LanB1 expression, which is ameliorated by Xbp1. The combination of LanB1 with Aβ and Xbp1^RNAi^, it resulted in an even greater toxicity compared to Aβ and Xbp1^RNAi^ alone. Conversely, overexpression of LanB1 significantly rescued the short lifespan of Aβ flies with Xbp1 knockdown (Fig. 7F), and there was no significant difference in lifespan between these rescue flies and Aβ-alone controls (*p* = 0.067, log rank). Thus, the ability of LanB1 to rescue Aβ toxicity was independent of Xbp1. Since LanB1 did not affect BiP levels in the context of neuronal Aβ, and since LanB1 could rescue Aβ toxicity independently of Xbp1, LanB1 must have acted independently of the IRE1α/XBP1s arm of the ER stress response pathway.

### 3.12 Proteins that are retained in the ER can rescue Aβ toxicity

**Fig. 8:**
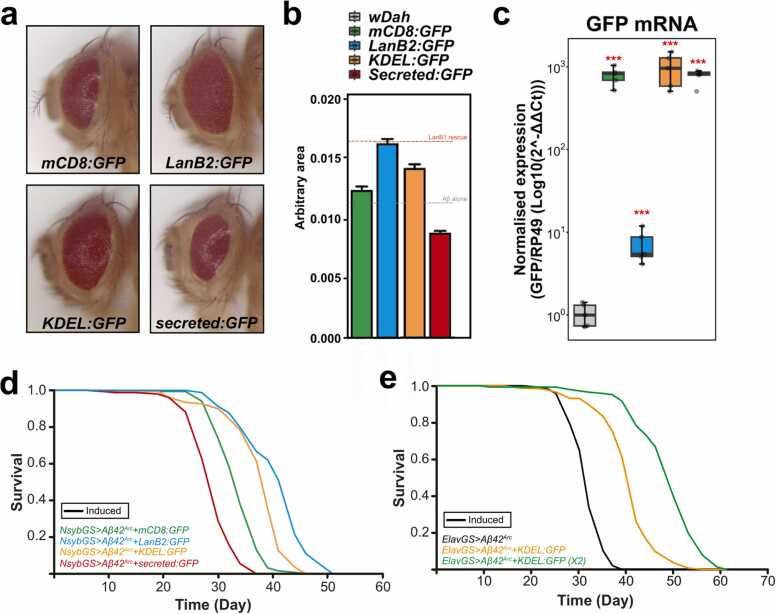
**Protein accumulation in the ER may be responsible for the rescue of Aβ toxicity**. (A and B) Co-expression of Aβ^X2^ with LanB2:GFP or KDEL:GFP rescued Aβ toxicity in the developing eye compared to mCD8:GFP controls, while secr:GFP exacerbated Aβ toxicity. (B) Quantification of eye sizes in (A). mCD8:GFP did not rescue the rough eye phenotype compared to Aβ^X2^ alone (see Supplementary Fig. 5). LanB2:GFP and KDEL:GFP significantly rescued eye size while secr:GFP significantly reduced eye size compared to mCD8:GFP controls (*p* < 0.0001; 1-way analysis of variances with Dunnett’s post-hoc test). Data are shown as mean ± SEM (n = 34–46 eyes measured per condition). For display purposes, 2 micrographs in (A) are the same as those in Supplementary Fig. 5. (C) *GFP* mRNA was significantly upregulated in all GFP-tagged conditions compared to the *ElavGS/w*^*Dah*^ control (*p* < 0.001; 1-way analysis of variances on log-transformed data; *p* values were adjusted using the Tukey method for multiple comparisons); n = 5 biological replicates per condition. The Y-axis is the log scale. (D) Survival curves of female flies induced to express Aβ^Arc^ via NsybGS. LanB2:GFP and KDEL:GFP rescued Aβ toxicity while secr:GFP exacerbated Aβ toxicity compared to mCD8:GFP controls (LanB2:GFP, *p* = 2.37 × 10^−26^; KDEL:GFP, *p* = 3.49 × 10^−17^; secr:GFP, *p* = 1.79 × 10^−19^; log rank). (E) One copy of KDEL:GFP significantly rescued Aβ^Arc^ toxicity (*p* = 1.15 × 10^−44^; log-rank test), while 2 copies of KDEL:GFP led to an even greater rescue (*p* = 9.18 × 10^−27^; log-rank test comparing 1 vs. 2 copies of KDEL:GFP). Genotypes: (A and B) *GMR*>*Aβ*^*X2*^*+ mCD8:GFP* ; *GMR*>*Aβ*^*X2*^*+ LanB2:GFP*; *GMR*>*Aβ*^*X2*^*+ KDEL:GFP*; *GMR*>*Aβ*^*X2*^*+ secr:GFP*, (C) *ElavGS/w^Dah^*; *ElavGS*>*mCD8:GFP*; *ElavGS*>*LanB2:GFP*; *ElavGS*>*KDEL:GFP; ElavGS*>*secr:GFP*. For all lifespan experiments, n = 150 flies per condition.

Since LanB1 overexpression resulted in intra-ER accumulation, we investigated whether overexpression of other proteins restricted to the ER could result in the same phenotype. To test this, we compared the effects of GFP localized to different cellular compartments, namely membrane-targeted mCD8:GFP, LanB2:GFP (laminin γ chain), which accumulates in the ER, KDEL:GFP which is retained in the ER, and secr:GFP which is secreted extracellularly, and measured their effect on Aβ^X2^ toxicity in the developing eye. As with LanB1 overexpression, expression of LanB2:GFP rescued Aβ toxicity (Fig. 8A and quantified in Fig. 8B) compared to mCD8:GFP controls. Expressing KDEL:GFP led to a rescue of eye size while secr:GFP exacerbated toxicity. As the level of rescue may be due to differing expression levels, we assessed the expression levels of each GFP-tagged protein using qPCR (Fig. 8C). mCD8:GFP, KDEL:GFP, and secr:GFP were expressed at levels ∼1000-fold higher compared to *ElavGS/w*^*Dah*^ control, but there was no statistical difference in gene expression between these GFP-tagged proteins. Therefore, the rescue of Aβ toxicity by KDEL:GFP was not due to its expression level since mCD8:GFP and secr:GFP, expressed at the same level, did not rescue Aβ toxicity. LanB2:GFP was also significantly elevated compared to control, though to a much lesser extent (∼8-fold) than the other GFP-tagged proteins. In adult neurons, LanB2 significantly rescued the short lifespan induced by Aβ toxicity (Fig. 8D and Supplementary Fig. 8B). Again, KDEL:GFP rescued Aβ^Arc^ toxicity and secr:GFP exacerbated Aβ^Arc^ toxicity (Fig. 8D). We observed a dose-dependent rescue of Aβ toxicity by KDEL:GFP with 2 copies of KDEL:GFP, leading to an even greater rescue than 1 copy (Fig. 8E). Thus, the retention of overexpressed proteins in the ER ameliorated Aβ toxicity. Overall, these results suggest that ER retention is a promising avenue of AD research.

### 3.13 Lamb1 also accumulates in the ER when overexpressed in mouse neural tissue

**Fig. 9:**
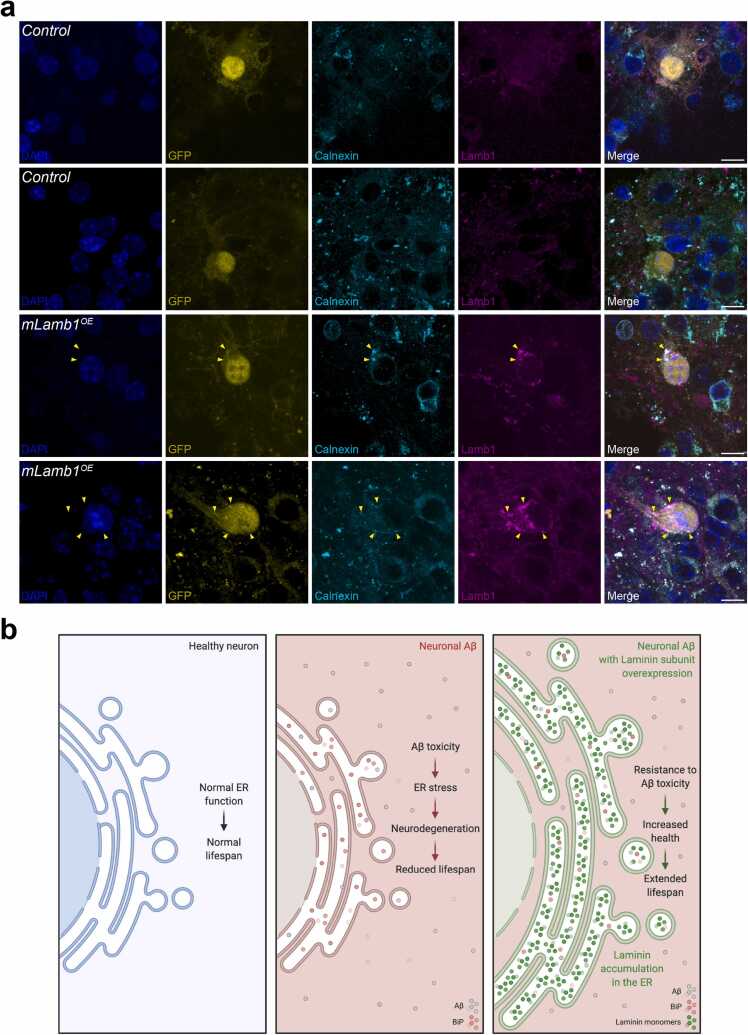
**Intra-ER retention of overexpressed Lamb1 is conserved in mouse brain tissue**. (A) Organotypic hippocampal slice cultures from 10-day-old mouse pups were incubated for 24 hours with lentivirus containing *mLamb1+*GFP or control lentivirus (GFP only), and then fixed 2-weeks later. Slices were then stained for Lamb1 and the ER marker, Calnexin. Endogenous GFP expression was used to identify successful transduction. DAPI labeled nuclei. The top 2 rows show examples of control lentivirus without *mLamb1* induction. The bottom 2 rows show examples of lentiviral *mLamb1* induction. Yellow arrowheads indicate areas of colocalization of Lamb1 and Calnexin. (B) Summary model of intra-ER laminin retention. Created with BioRender.com. Scale bar, 10 µm. Abbreviations: Aβ, Amyloid β; ER, endoplasmic reticulum.

Disruption of laminin subunit production results in intracellular accumulation of the remaining subunits (Petley-Ragan et al., 2016). To our knowledge, however, there are no published data examining the overexpression of Laminin subunits and their expression pattern in mammalian models. We used ex-vivo mouse organotypic hippocampal slice cultures to transduce mouse Lamb1 using an engineered lentivirus. Organotypics were generated from P10 wild-type (C57/BL6J) mouse pups and incubated with mouse Lamb1 (mLamb1 + GFP) recombinant lentivirus or control recombinant lentivirus (GFP only). Cultures were transfected with the virus at 14 days *in vitro* and fixed after a further 14 days. Overexpression of mLamb1 resulted in marked intracellular accumulation compared to controls and did not result in a uniform cytoplasmic distribution, indicating compartmentalization (Fig. 9A). Lamb1 colocalized with the ER marker Calnexin in some areas, indicating retention of Lamb1 in the ER, though there were also areas in these cells with no overlap. Therefore, the ER retention of the overexpressed laminin β chain is a conserved process.

## 4 Discussion

Here, we showed that ectopic, neuronal overexpression of laminin (and collagen IV) monomers provided robust protection in an *in vivo Drosophila* model of AD, and that ectopic overexpression of LanB1 resulted in ER retention in these neurons. Overexpression of mouse Lamb1 in *ex vivo* mouse organotypic hippocampal slice cultures also resulted in ER retention of these monomers, highlighting a conserved process. In the fly, LanB1 rescued Aβ toxicity (but not polyQ or (G_4_C_2_)_36_ toxicity) without reducing Aβ levels (soluble or insoluble) and without affecting Aβ secretion into the extracellular milieu. LanB1 also rescued Aβ toxicity in combination with other laminin subunits and a collagen IV subunit and acted independently of the IRE1α/XBP1s ER stress response branch. Finally, the expression of other proteins targeted for retention in the ER could significantly rescue Aβ toxicity. Specifically, we observed a dose-dependent rescue of Aβ toxicity by ER-retained GFP with more copies, resulting in a larger amelioration of toxicity. Overall, we have described a novel mechanism whereby ER retention of proteins, typically detrimental to cellular health, is a potential therapeutic target for AD (illustrated in Fig. 9B).

Regrettably, the transduction efficiency of our mLamb1 lentivirus was significantly low, impeding the progression of further experiments. For instance, we were unable to assess whether the overexpression of mLamb1 affected APP/Aβ. Consequently, we could not validate the hypothesis concerning the conserved protective impact of mLamb1 protein retention against Aβ toxicity. Nevertheless, we maintain the view that the proof-of-principle experiment, which demonstrated the accumulation of mLamb1 in the ER upon overexpression and underscored its conservation across species, holds substantial relevance.

For nearly 3 decades, Aβ has been one of the major targets for AD therapy with most major clinical trials aiming to reduce or detoxify Aβ (Selkoe and Hardy, 2016). Similarly, most studies in *Drosophila* showing rescue of the Aβ toxicity appear to work, at least partially, via the reduction of Aβ levels (Finelli et al., 2004, Marcora et al., 2017, Sekiya et al., 2017, Sofola et al., 2010). Previous studies in both flies and humans, however, have demonstrated that Aβ load can be uncoupled from toxicity (Arboleda-Velasquez et al., 2019, Casas-Tinto et al., 2011, Niccoli et al., 2016). Intriguingly, LanB1 was able to substantially rescue Aβ toxicity without altering levels of Aβ, either soluble or insoluble, and also without reducing the secretion of Aβ into the extracellular milieu, indicating that LanB1 expression increased neuronal resistance to Aβ toxicity. This result also suggests that, since LanB1 accumulated in the ER, the beneficial effect of LanB1 overexpression may also occur in the ER. Further work examining the intracellular and extracellular availability of LanB1 may inform some of the debate surrounding intracellular versus extracellular Aβ toxicity (Strobel, 2011).

Collagen VI rescues Aβ toxicity by sequestering Aβ into large aggregates in the extracellular milieu (Cheng et al., 2009). Hence, LanB1 could have promoted the formation of Aβ fibrils from the more toxic oligomeric form. However, we observed no difference in the levels of soluble or insoluble Aβ levels with LanB1 co-expression, indicating that LanB1 was not altering the ratio of soluble/insoluble Aβ. It remains to be seen whether Aβ is binding to LanB1, or if LanB1 is sequestering toxic oligomeric Aβ into less toxic structures. Crucially, a rescue of Aβ toxicity was also observed with Cg25C, LanA, LanB2, and KDEL:GFP, the last of which likely does not bind Aβ, indicating mechanisms other than Aβ sequestration by LanB1.

Since glia are the other major cell type in the adult fly brains and can produce laminins, we determined if LanB1 overexpression in these cells could also rescue Aβ toxicity. We could not verify this, however, as we did not observe Aβ toxicity using GliaGS, and co-expression of LanB1 also had no effect. This result was consistent with recent studies that found glial Aβ was less toxic than neuronal Aβ despite having a much higher brain load than those produced by neurons (Jonson et al., 2018, Marcora et al., 2017).

ER stress has been implicated in the progression of AD (Gerakis and Hetz, 2017), although the precise mechanisms that underlie protein misfolding contributions to AD pathogenesis are still unclear (Scheper and Hoozemans, 2015). Here, we found that BiP was not affected at the mRNA/protein level by LanB1, despite the rescue of Aβ toxicity. It is possible that BiP expression was at the upper limit and LanB1 could not increase BiP expression further. Regardless, it is likely that BiP was chronically high throughout the adult lifespan, which is detrimental to neuronal health when co-expressed with Aβ (Sekiya et al., 2017). Intriguingly, BiP may be a molecular chaperone of laminin assembly in the ER (Kumagai and Kitagawa, 1997), which may indicate that LanB1 overexpression sequesters BiP from having toxic effects when upregulated chronically.

The importance of Xbp1 in the response to Aβ is evident with the knockdown of Xbp1, which has been shown to enhance Aβ toxicity (Casas-Tinto et al., 2011, Marcora et al., 2017). However, LanB1 could rescue the enhanced toxicity of Aβ+Xbp1^RNAi^ in adult neurons, indicating that LanB1 could act independently of Xbp1. Conversely, we found that LanB1 expression further enhanced the toxicity of Aβ+Xbp1^RNAi^ in the developing eye, while LanB1 and Xbp1^RNAi^ without Aβ also resulted in a rough eye phenotype, indicating LanB1 expression in the developing eye has the potential to be toxic and Xbp1 is protecting against this toxicity. The discrepancy in results could be explained by the context-dependent nature of the different assays. The eye degeneration phenotype is based on developmental expression in a variety of dividing cell types in the developing eye while the lifespan results rely on adult-onset expression in terminally-differentiated neurons.

Knocking down Xbp1 increased Aβ protein levels while overexpression reduced Aβ protein levels (Marcora et al., 2017). However, we found no difference in Aβ levels with LanB1 co-expression, further indicating that the beneficial effects are independent of Xbp1. Further work analyzing epistatic interactions between LanB1 overexpression and the ER stress response branches will be necessary to deduce the mechanism underlying the rescue of Aβ toxicity.

LanA also rescued Aβ toxicity but not to the same extent as LanB1, but this was likely due to the transgene being expressed at a much lower level compared to LanB1:RFP and Cg25C:RFP transgenes. Previous studies have shown that the laminin α-chain can be secreted as a monomer without the requirement for heterotrimerization (Kumagai et al., 1997, Yurchenco et al., 1997). Thus, an alternative hypothesis is that some LanA is secreted from the ER in monomeric form and so the ER may not accumulate as many LanA subunits and hence has a reduced ability to rescue Aβ toxicity. If this were the case, it would be expected that overexpression of the other laminin α-chain (wb) might exhibit the same degree of Aβ rescue as LanA. Collagen IV also forms an obligate heterotrimer with 2 chains of Cg25C and 1 Vkg chain. Similar to LanA, Vkg can be secreted as a monomer (Pastor-Pareja and Xu, 2011), and it is thought that Cg25C requires Vkg for secretion (like LanB1/LanB2).

Laminin or collagen upregulation is considered detrimental in most contexts (Patton, 2000, Yu et al., 2007) and especially in relation to cancer invasiveness (Huang et al., 2019, Rousselle and Scoazec, 2020). Indeed research examining disease-causing mutations in laminin and collagen has focused on defective ECM-receptor signaling as the underlying cause of pathology (Huang et al., 2019, Yu et al., 2007). Other studies have focused on receptor-independent effects of laminin and collagen loss and have found that mutations triggered ER stress, potentially due to defective secretion of remaining subunits (Chen et al., 2013, Murray et al., 2013, Petley-Ragan et al., 2016). A case study of a patient with porencephaly (cystic brain lesions) carrying a collagen IV α2 mutation, which caused intracellular accumulation of the COL4A2 chain, found ER stress and UPR activation, which could be rescued by chemical chaperone treatment (Murray et al., 2013). Interestingly, the patient’s father carrying the same allele also displayed basement membrane defects but no disease symptoms, indicating that intracellular accumulation of COL4A2 rather than extracellular effects led to disease (Murray et al., 2013).

Our hypothesis that laminin-/collagen-subunit retention in the ER in neurons is beneficial is augmented by the finding that ER-retained GFP (KDEL:GFP) also robustly reduced Aβ toxicity in a dose-dependent manner. Interestingly, GFP targeted for extracellular secretion (secr:GFP) exacerbated Aβ toxicity. It is possible that extracellular GFP is degraded by glia and thus reduces the capacity of glia to clear Aβ, leading to increased toxicity. In agreement with our finding that secr:GFP exacerbated Aβ toxicity, Fernandez-Funez et al. (2016) showed that eyes of GMR>Aβ+secr:GFP flies were smaller when compared to GMR>Aβ+LacZ. In addition, prion protein, a molecule located extracellularly, exacerbated Aβ toxicity when co-expressed, and caused massive Aβ deposition in the brain (Younan et al., 2018).

We have discovered that increased expression of ER-retained proteins, typically seen as detrimental, rescued Aβ toxicity in neurons, and could be a new therapeutic avenue for AD research.

Submission declaration and verification

The work described has not been published previously, it is not under consideration for publication elsewhere, and its publication has been approved by all authors. If accepted, the work will not be published elsewhere in the same form, in English or in any other language, including electronically without the written consent of the copyright-holder.

## Author contributions

Conceived and designed the experiments: JHC, LP. Performed the experiments: JC, LM, SA, SJM, SM, MCD, AR, NSW, MLA, MA, CD. Analyzed the data: JHC. Contributed reagents/materials/analysis tools: JHC, TLSJ, LP. Wrote the paper: JHC, LP.

## Disclosure statement

None.
