# Supplementary material for: Protein retention in the endoplasmic reticulum rescues Aβ toxicity in Drosophila
Source: Neurobiol Aging. 2023 Dec;132:154–74. doi: 10.1016/j.neurobiolaging.2023.09.008 (PMC10940166; doi:10.1016/j.neurobiolaging.2023.09.008)
Supplement: Supplementary file 1 — Supplementary material [file mmc1.docx]

**Supplementary Figures**


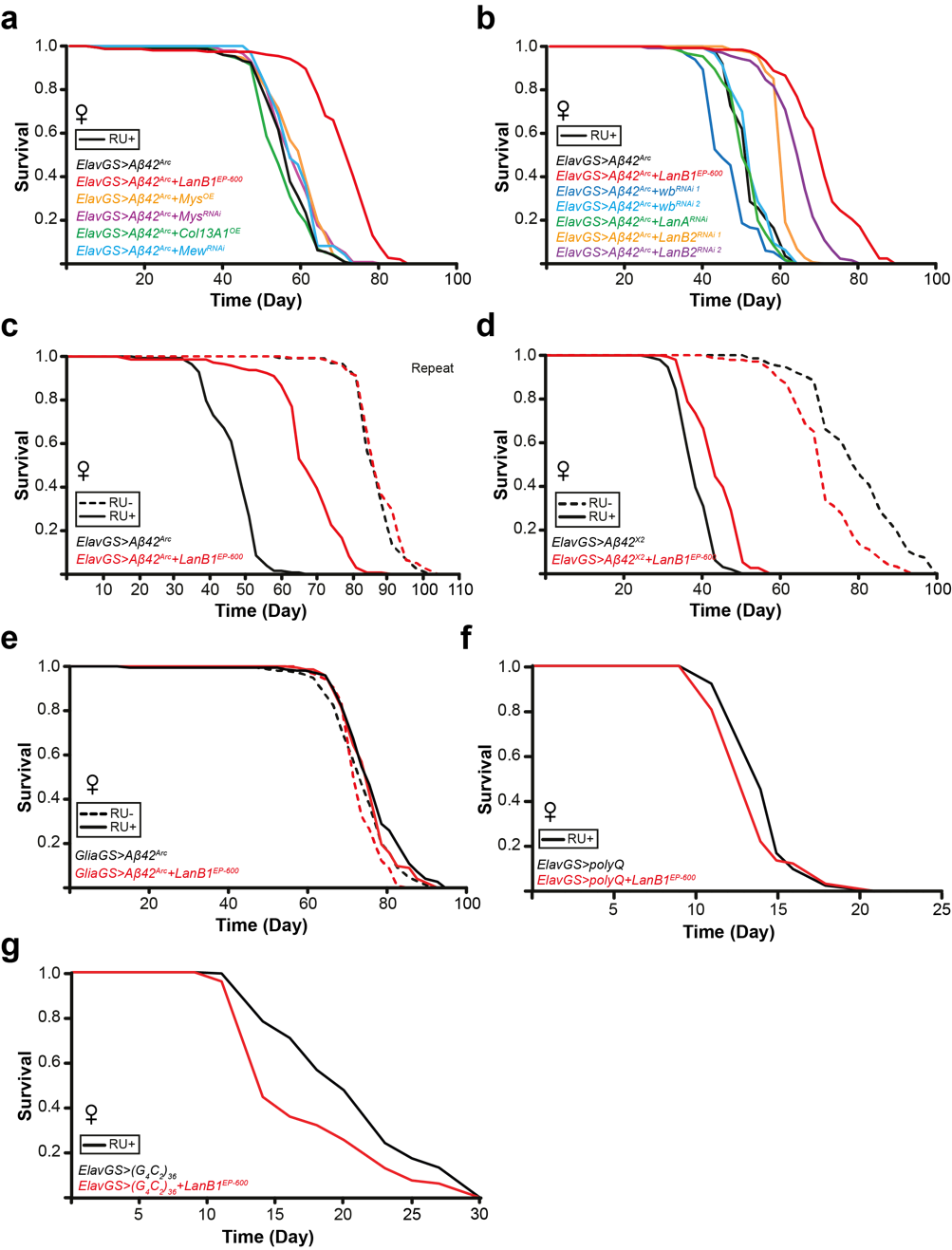


**Supplementary Fig. 1** Uncovering a role for LanB1 in the amelioration of Aβ toxicity. **a** ECM-related transgenic lines were crossed with flies expressing pan-neuronal Aβ^Arc^ with the ElavGS driver. LanB1 co-expression significantly rescued the short-lifespan phenotype compared to the induced Aβ^Arc^-alone control (p = 2.86 x 10^-44^; log rank test). **b** Additional Laminin transgenic lines were examined for their effect on Aβ toxicity. LanB1 co-expression significantly rescued toxicity compared to induced controls (p = 1.08 x 10^-54^; log rank test). Both independent LanB2 RNAi lines also exhibited significant rescue of Aβ toxicity (LanB2^RNAi 1^ p = 7.94 x 10^-35^; LanB2^RNAi 2^ p = 4.13 x 10^-41^ log rank test). **c** Induction of Aβ^Arc^ significantly (p = 8.34 x 10^-67^; log rank test) shortened lifespan compared to uninduced controls. LanB1 and Aβ^Arc^ co-expression resulted in a significant rescue (p = 1.48 x 10^-56^; log rank test) of the short-lived phenotype. **d** Expression of two copies of wildtype Aβ_42_ (Aβ_­_^X2^) in neurons. Aβ_­_^X2^ significantly (p = 4.52 x 10^-68^; log rank test) shortened lifespan compared to uninduced controls. There was a significant difference between uninduced control lines (p = 4.00 x 10^-12^; log rank test), but in the reverse direction to the rescue. LanB1 and Aβ_­_^X2^ co-expression resulted in a significant rescue (p = 6.47 x 10^-15^; log rank test). **e** Glial expression of Aβ was not toxic. Using a glial GeneSwitch driver (GliaGS), flies with adult-onset glial Aβ expression had slightly extended lifespan compared to uninduced controls (Aβ p = 0.0074; Aβ+LanB1 p = 0.0074; log rank test). LanB1 co-expression did not affect this extension (comparison in induced conditions p = 0.14; log rank test). **f** LanB1 did not rescue polyQ toxicity. Induction of polyQ is toxic and results in shortened lifespan. LanB1 and polyQ co-expression resulted in a significant reduction in lifespan compared to polyQ-alone controls (p = 0.018; log rank test). **g** LanB1 did not rescue C9orf72 hexanucleotide repeat expansion toxicity. Induction of (G_4_C_2_)_36_ is toxic and results in shortened lifespan. LanB1 and (G_4_C_2_)_36_ co-expression resulted in a significant reduction in lifespan compared to (G_4_C_2_)_36_-alone controls (p = 8.49 x 10^-07^; log rank test). For lifespan experiments n = 150 flies per condition.


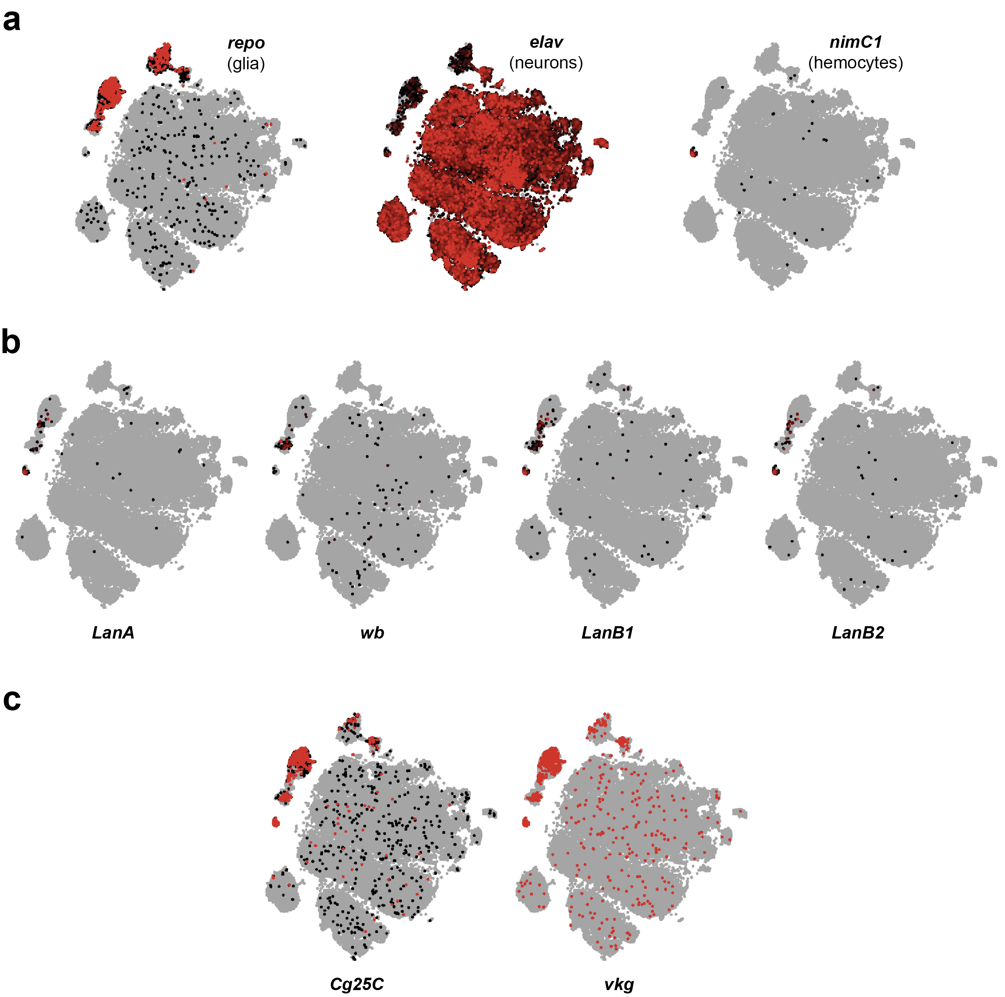


**Supplementary Fig. 2** Single-cell transcriptome atlas of the adult *Drosophila* brain shows that laminin- and collagen IV-subunit expression is restricted to haemocytes and a subset of glial cells. **a** Cell types in the brain can be distinguished by specific gene expression. Glial cells are specified by *repo*, neurons by *elav*, and haemocytes by *nimC1* expression. **b** All four laminin subunits in *Drosophila* (LanA, wb, LanB1, LanB2) are expressed in haemocytes and a subset of glial cells but are not substantially expressed in neurons. **c** The two collagen IV subunits (Cg25C, vkg) are highly expressed in haemocytes and glial cells with some neuronal expression. Atlas images were obtained by gene name searches in *SCope*, the vizualisation and analysis tool from the Aerts lab (<http://scope.aertslab.org/>).

**
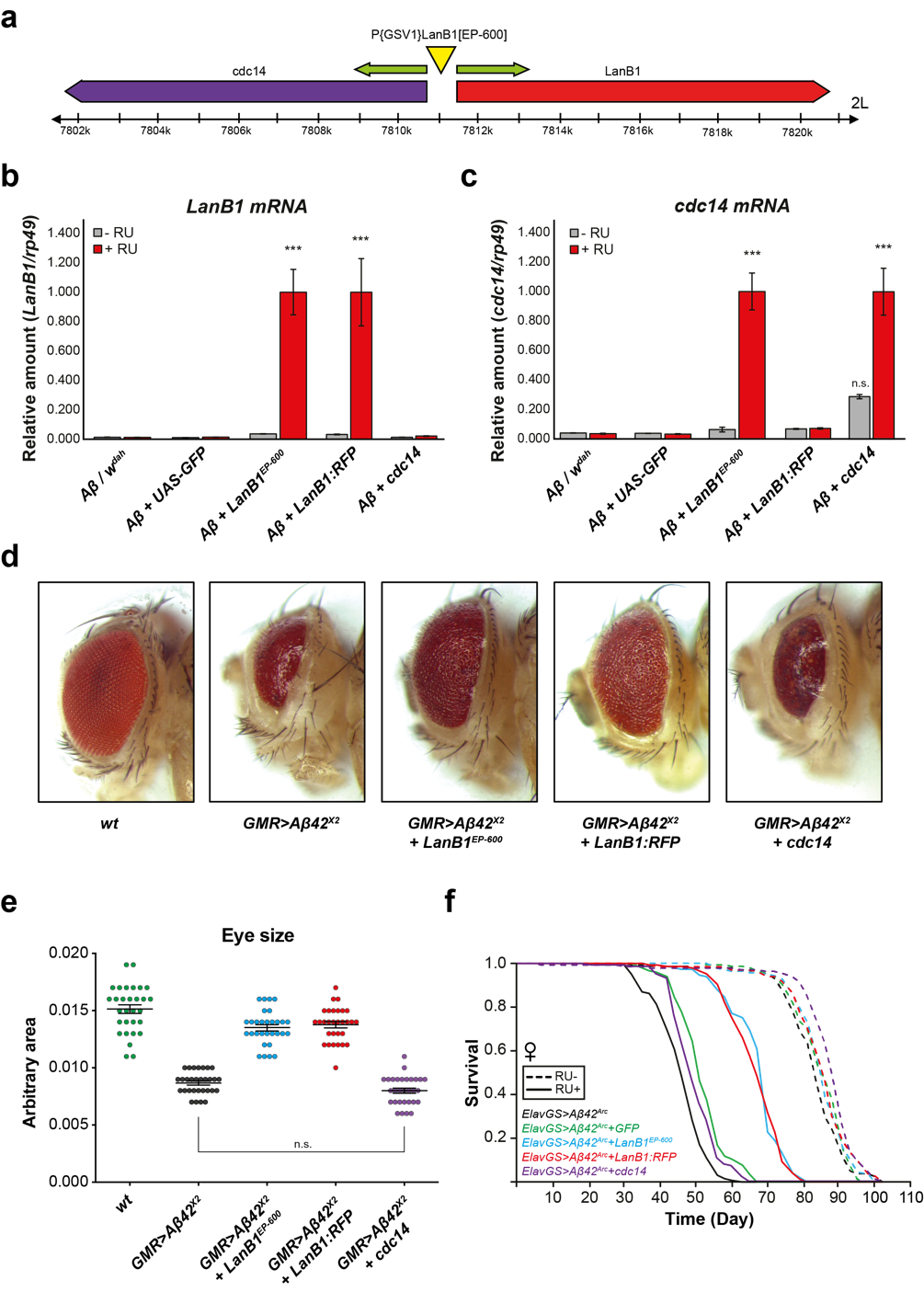
**

**Supplementary Fig. 3** Expression of *LanB1* and not *cdc14*, an adjacent gene in the opposite orientation, rescued Aβ toxicity. **a** Schematic of the LanB1^EP-600^ locus located 548 bases upstream of the 5' UTR of the LanB1 gene. The P{GSV1} element contains UAS sequences at both ends oriented outwards. **b** *LanB1* was significantly upregulated in the heads of UAS-LanB1^EP-600^ and UAS-LanB1:RFP flies compared to uninduced controls (p < 0.0001; one-way ANOVA with Tukey’s post-hoc test). **c** *Cdc14* was significantly up-regulated in the heads of UAS-LanB1^EP-600^ and in UAS-cdc14 (cdc14^EY10303^) heads compared to uninduced controls (p < 0.0001; one-way ANOVA with Tukey’s post-hoc test). Cdc14 up-regulation was not observed in UAS-LanB1:RFP heads. Mild up-regulation in uninduced UAS-cdc14 heads may be due to compensatory up-regulation as the cdc14^EY10303^ P-element is inserted 395 bases into the 5’ UTR of cdc14. Relative qPCR values were normalized to *rp49* expression. Data are shown as mean ± SEM (n = 4 biological replicates per condition). **d** Co-expression of Aβ_­_^X2^ with LanB1 (via UAS-LanB1^EP-600^ and UAS-LanB1:RFP) using the eye-specific GMR-GAL4 driver rescued most of the size and organisation of the eye. Cdc14 did not rescue Aβ_­_^X2^ toxicity. For display purposes, three micrographs in **d** are the same as those in **Fig 1a**. **e** Quantification of eye sizes in **d**. There was no significant difference in eye size between Aβ_­_^X2^ alone or with cdc14 co-expression. LanB1 (via UAS-LanB1^EP-600^ and UAS-LanB1:RFP) significantly rescued eye size (p < 0.0001; one-way ANOVA with Tukey’s post-hoc test). Data are shown as mean ± SEM (n = 30 eyes measured per condition). **f** The considerable rescue from Aβ toxicity by LanB1^EP-600^ was due to LanB1 and not cdc14. Although co-expression of cytoplasmic GFP (BDSC #1521) or cdc14 with Aβ^Arc^ led to small but significant rescues of lifespan compared to Aβ^Arc^ alone controls (cytoplasmic GFP, p = 2.44 x 10^-12^; cdc14, p = 2.64 x 10^-6^ log rank test), LanB1 and Aβ^Arc^ co-expression (via UAS-LanB1^EP-600^ and UAS-LanB1:RFP) exhibited equivalently large rescues of Aβ toxicity (LanB1^EP-600^, p = 1.39 x 10^-61^; LanB1:RFP, p = 2.85 x 10^-62^ log rank test). n = 150 flies per condition.


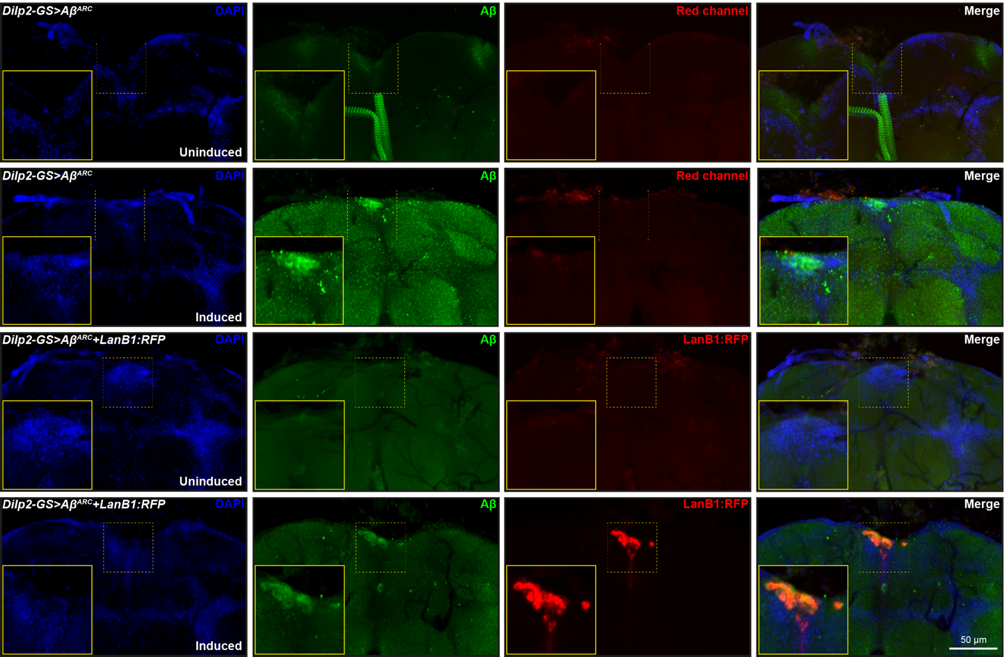


**Supplementary Fig. 4** Inducible expression of Aβ and LanB1 in Dilp2 neurons using the Dilp2-GS driver. Without RU induction, there was no induction of Aβ and/or LanB1 expression. When fed RU, Aβ and LanB1 were expressed. Representative confocal fluorescence z projections taken at 20x magnification of whole brains from 21-day-old female flies stained with Aβ (6E10 – green) and DAPI (blue). Yellow box inset shows a higher magnification of the Dilp2 neuron cluster area in the dorsal brain. Endogenous fluorescence (i.e. without staining) of LanB1:RFP is shown. Genotypes: *Dilp2-GS>Aβ^Arc^*; and *Dilp2-GS>Aβ^Arc^ + LanB1:RFP*. Scale bar, 50 μm.


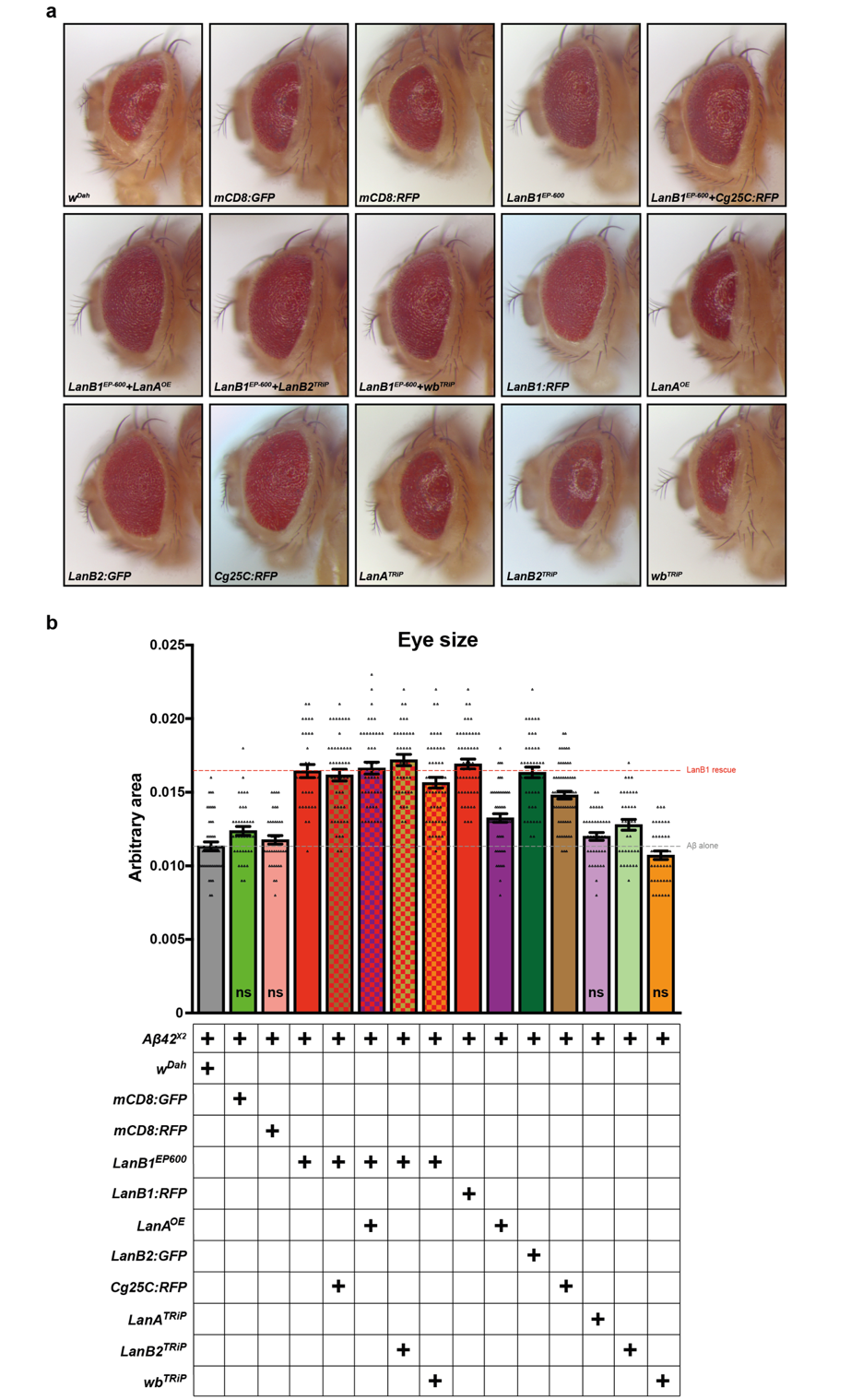


**Supplementary Fig. 5** Laminin-induced rescue of Aβ toxicity in the developing eye is not affected by modulation of other laminin subunits. **a** All flies shown expressed Aβ_­_^X2^ using GMR-GAL4. The degree of rescue from LanB1 over-expression (via UAS-LanB1^EP-600^ and UAS-LanB1:RFP) was not affected by expression of the indicated genes. **b** Quantification of eye sizes in **a**. Eye size for each condition was quantified and compared to the *ElavGS>Aβ_­_^X2^/w^Dah^* control. Over-expression or knockdown of other Laminin subunits in combination with LanB1 had no significant effect on the rescue. Conditions marked ‘ns’ were not significantly different compared to controls, while the other lines were significantly different (one-way ANOVA with Tukey’s post-hoc test). Plus (**+**) symbol indicates presence of labelled transgene. Bars containing red indicate presence of a LanB1 over-expression line. Dashed grey line indicates the Aβ control, while dashed red line indicates the LanB1-rescue level. ‘TRiP’ indicates an RNAi transgene. Data are shown as mean ± SEM (n = 34-62 eyes measured per condition).

**
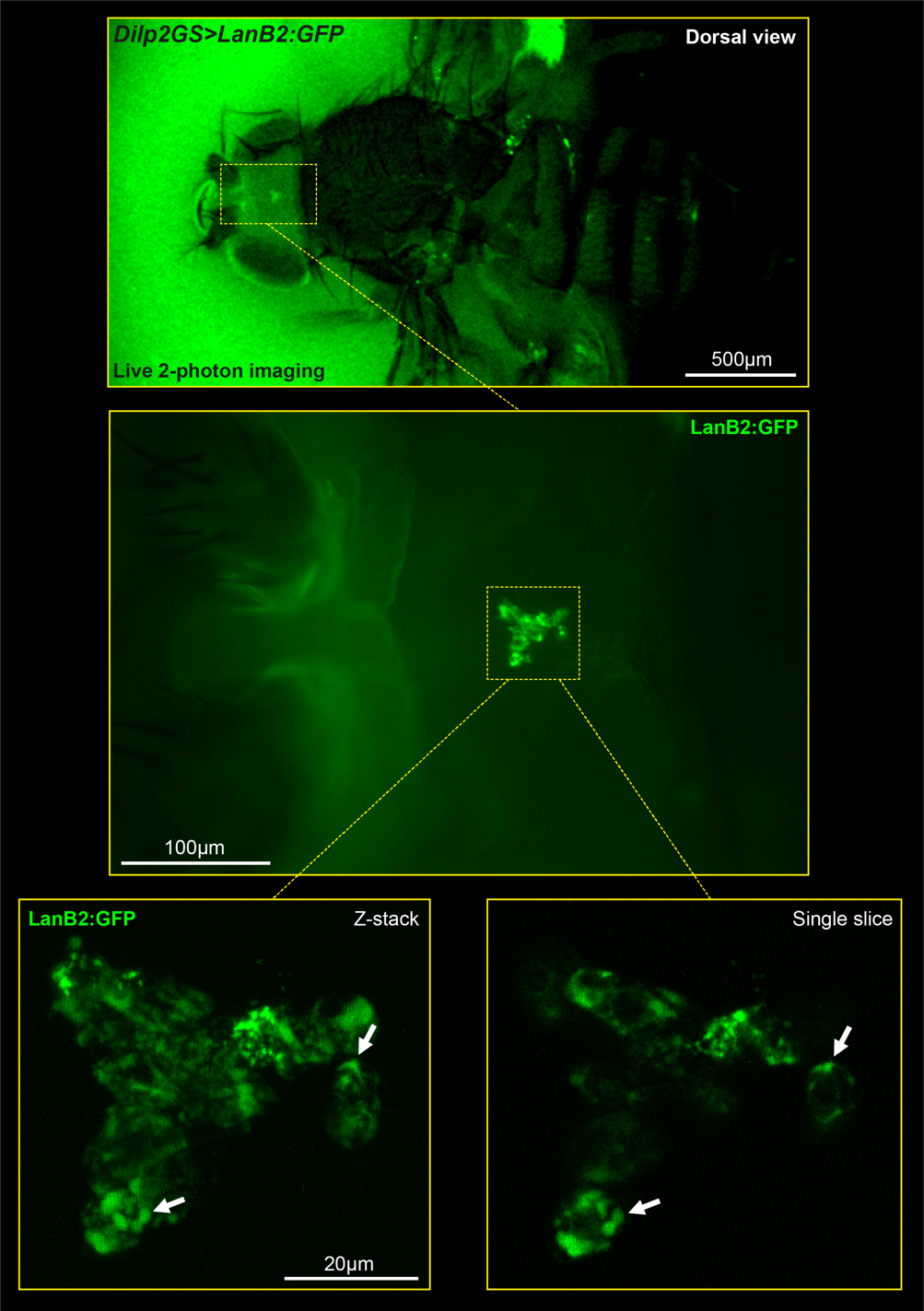
**

**Supplementary Fig. 6** Live 2-photon imaging of a fly producing LanB2:GFP in Dilp2 neurons. LanB2 accumulated intracellularly in discrete compartments. *Top*, dorsal view of fly with cuticle removed to visualize the brain. To prevent dehydration, saline gel was added with a small coverslip on top. *Middle*, closer dorsal view of live fly brain. *Bottom left*, z-projection of the cell bodies of the Dilp2 neurons expressing LanB2:GFP. *Bottom right*, single slice of these cell bodies showing LanB2 accumulated in discrete cellular compartments and not diffusely in the cytoplasm. Arrows show discrete intracellular accumulations of LanB2:GFP. Genotype: *Dilp2-GS>LanB2:GFP*. In all images anterior is left, posterior is right. Scale bar, top 500 μm, middle 100 μm, bottom, 20 μm.


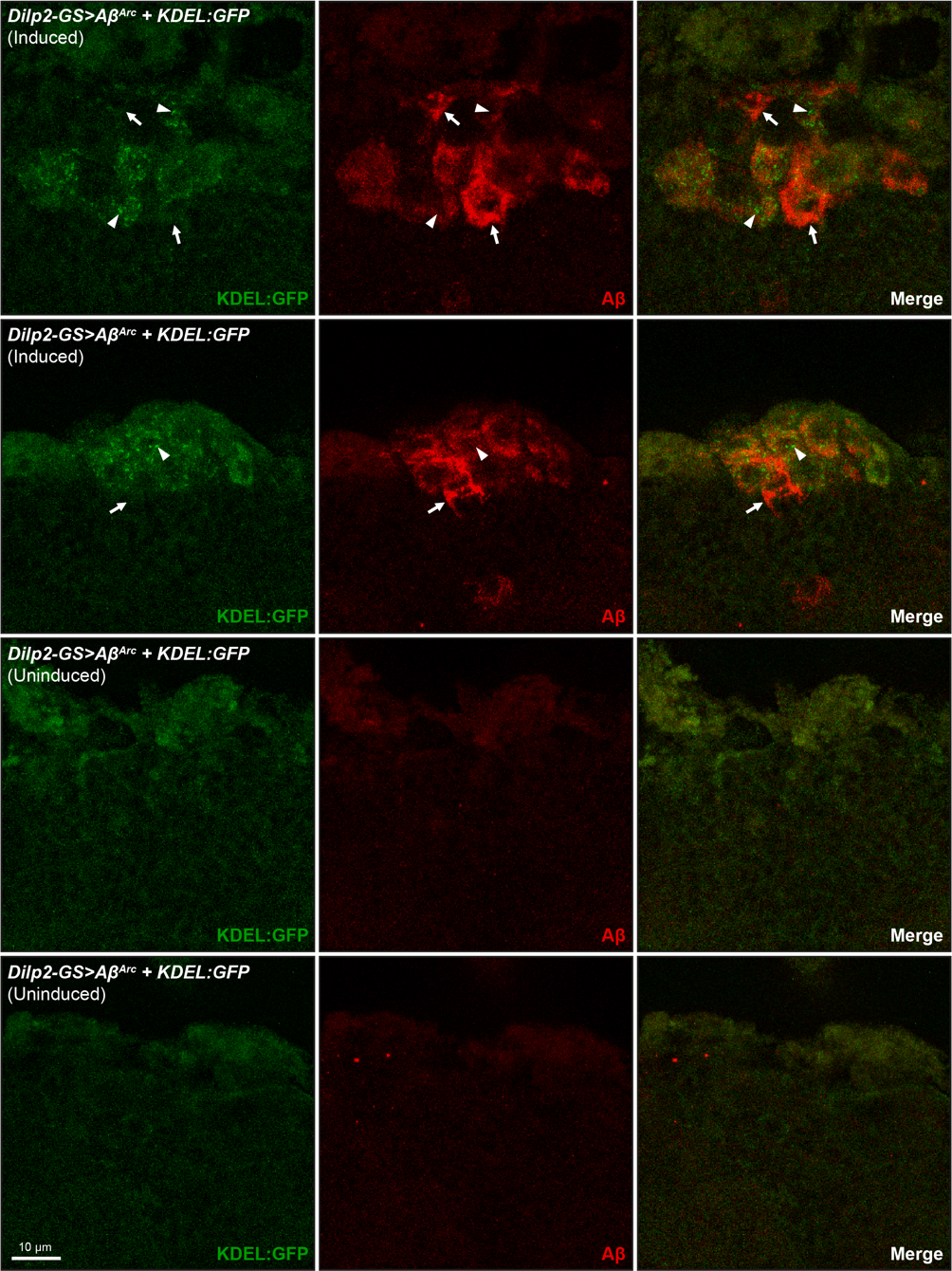


**Supplementary Fig. 7** Aβ does not accumulate in the ER. Inducible expression of Aβ and the ER marker, KDEL:GFP, in Dilp2 neurons using the Dilp2-GS driver. *Top two rows*, when fed RU, Aβ and KDEL:GFP were present in the cell bodies of Dilp2 neurons. Arrows highlight areas with no overlap of Aβ and KDEL:GFP. Arrowheads highlight punctate areas of ER with no corresponding Aβ expression. *Bottom two rows*, without RU486 induction, there was no induction of Aβ and/or KDEL:GFP expression. Representative confocal fluorescence z projections taken at 63x magnification from 21-day-old female flies stained with Aβ (6E10 – green). Endogenous fluorescence (i.e. without staining) of KDEL:GFP is shown. Genotype: *Dilp2-GS>Aβ^Arc^ + KDEL:GFP*. Scale bar, 10 μm.


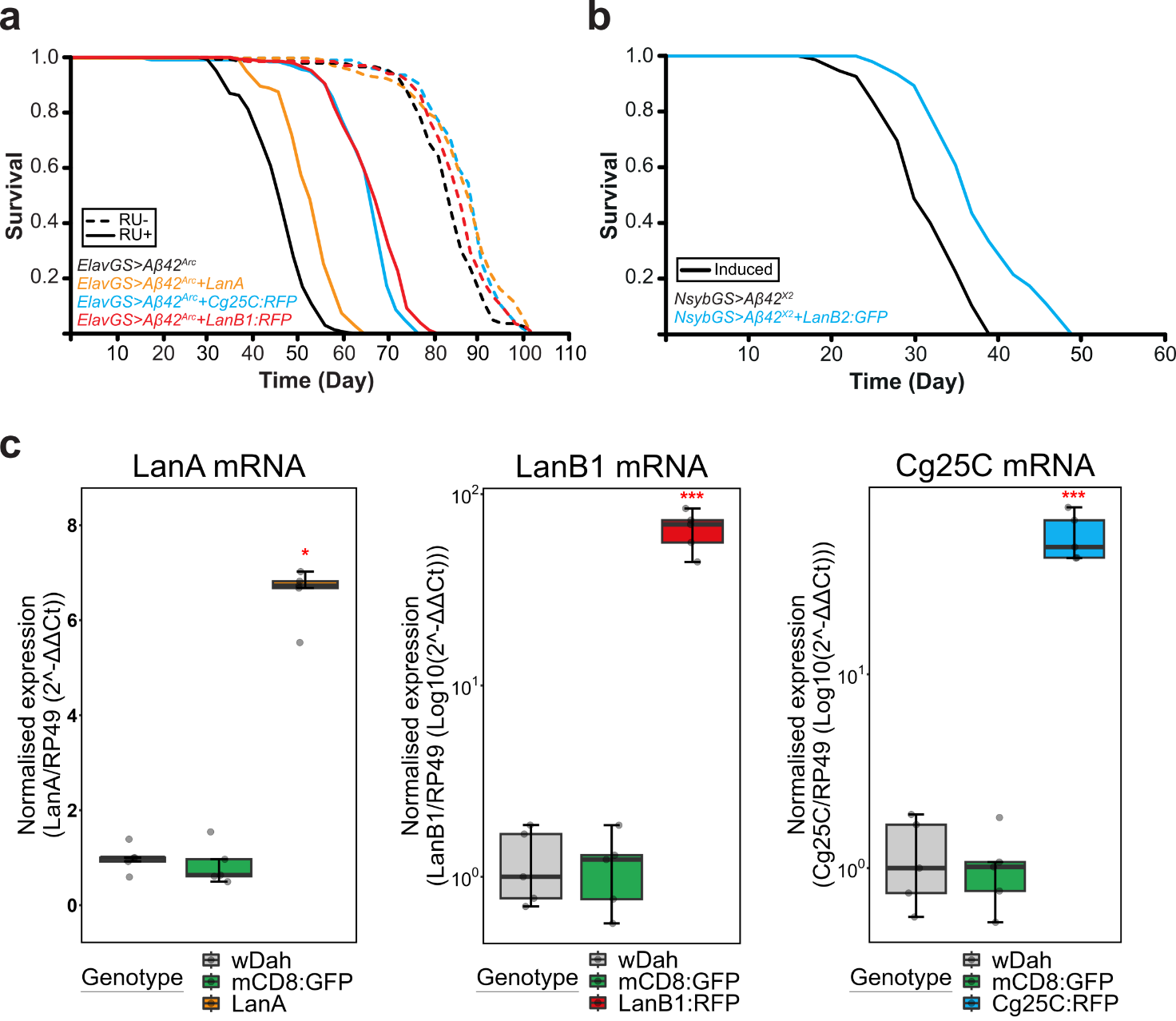


**Supplementary Fig. 8** Laminin γ-chain (LanB2) also rescued Aβ toxicity. **a** LanB1, Cg25C, and LanA rescued Aβ toxicity (LanB1, p = 2.85 x 10^-62^; Cg25C, p = 3.43 x 10^-63^; LanA, p = 1.58 x 10^-17^; log rank vs *ElavGS>Aβ^Arc^* alone). There were significant extensions of lifespan in the uninduced controls (LanB1, p = 0.036; Cg25C, p = 7.50 x 10^-06^; LanA, p = 7.33 x 10^-06^; log rank vs *ElavGS>Aβ^Arc^* alone). **b** LanB2 and Aβ_­_^X2^ co-expression using NsybGS resulted in a significant rescue (p = 1.00 x 10^-20^; log rank test) compared to induced Aβ_­_^X2^ controls. **c** LanA, LanB1, and Cg25C mRNA was significantly upregulated in all respective overexpression conditions compared to the *ElavGS/w^Dah^* control (LanA, p < 0.05; Kruskal-Wallis test, p values were adjusted using the Holm method for multiple comparisons; LanB1 and Cg25G, p < 0.0001; one-way ANOVA on log-transformed data, p values were adjusted using the Tukey method for multiple comparisons). n = 5 biological replicates per condition. Y-axis is log scale for LanB1 and Cg25C data. Genotypes: **c**, *ElavGS/w^Dah^*; *ElavGS>mCD8:GFP*; *ElavGS>LanB1:RFP*; *ElavGS>Cg25C:RFP*. For all lifespan experiments n = 150 flies per condition.
